# Negative Eigenvalue Estimates for the 1D Schrödinger Operator with Measure-Potential

**DOI:** 10.1007/s00023-025-01549-z

**Published:** 2025-02-17

**Authors:** Robert Fulsche, Medet Nursultanov, Grigori Rozenblum

**Affiliations:** 1 Institut für Analysis, Leibniz Universität Hannover, Welfengarten 1, 30167 Hannover, Germany; 2 Department of Mathematics and Statistics, University of Helsinki, Helsinki, Finland; 3 Institute of Mathematics and Mathematical Modeling, Almaty, Kazakhstan; 4 Chalmers University of Technology, Gothenburg, Sweden

**Keywords:** Primary 47A75, 34L15, Secondary 34E15

## Abstract

We investigate the negative part of the spectrum of the operator $-\partial ^2 - \mu$ on $L^2(\mathbb {R})$, where a locally finite Radon measure $\mu \ge 0$ serves as a potential. We obtain estimates for the eigenvalue counting function, for individual eigenvalues and estimates of the Lieb–Thirring type. A crucial tool for our estimates is Otelbaev’s function, a certain average of the measure-potential $\mu$, which is used both in the proofs and the formulation of most of the results.

## Introduction

The paper is devoted to obtaining negative spectrum estimates, in particular, Lieb–Thirring type estimates, for Sturm–Liouville operators with Radon measure serving as potential.

### Eigenvalue Estimates

Eigenvalue estimates for Schrödinger type operators are a classical topic in analysis. For Schrödinger operators of the form

1.1 $$\begin{aligned} \textbf{H}=-\Delta -V(x), \quad x\in \mathbb {R}^{\textbf{N}}, \end{aligned}$$

with the ‘potential’ *V*(*x*) tending to zero at infinity, there are numerous results concerning the number of negative eigenvalues, the sum of their powers and their distribution (when there are infinitely many of them). A huge, but still not exhausting, bibliography can be found in the recent book [15]. Of special interest is the Lieb–Thirring inequality [20], relating the spectral properties of the Schrödinger operator and properties of the corresponding classical Hamiltonian. It has, in its most classical case, the form

1.2 $$\begin{aligned} \textrm{LT}_{\gamma }(\textbf{H})\equiv \sum |\lambda _\nu (\textbf{H})|^\gamma \le C\int _{\mathbb {R}^\textbf{N}}V_+(x)^{\frac{\textbf{N}}{2}+\gamma }dx, \end{aligned}$$

where $\lambda _\nu (\textbf{H})$ are the negative eigenvalues and $C=C_{\gamma ,\textbf{N}}$ is a constant not depending on *V*. The inequality (1.2) holds for $\gamma \ge 0$ if $\textbf{N}\ge 3$ (for $\gamma =0$, it is called the CLR inequality), for $\gamma >0$ if $\textbf{N}=2$, and for $\gamma \ge \frac{1}{2}$ if $\textbf{N}=1$.

Among many possible generalizations and versions of eigenvalue inequalities, one line of research consists in finding versions of these estimates for the case when the potential *V*(*x*), classically supposed to be a function on $\mathbb {R}^\textbf{N}$, is, in fact, more singular, e.g., a Radon measure on $\mathbb {R}^\textbf{N}$. As a special case, one can consider measures which are singular with respect to the Lebesgue measure.

In physical and mathematical literature, such singular potentials have attracted considerable interest. A huge bibliography can be found in [1, 2, 5, 7–9, 13, 19, 22, 30] and many more. Of course, the one-dimensional case was studied in most detail.

As it concerns the quantitative spectral analysis for general measures serving as a potential, one of the complications is the absence of a quasi-classical pattern which usually hints for the expression entering into the eigenvalue inequalities and asymptotics—compared, say, with (1.2), where both the dependence on the potential *V* and even the best possible coefficient in the estimate are governed by certain quasi-classical heuristics. So, although the qualitative spectral analysis of such Schrödinger operators was satisfactory, the question on spectral estimates was rather unexplored.

### Singular Potentials

Probably the first rigorous quantitative spectral study involving singular potentials was [14], where, for a potential supported on a hyperplane in $\mathbb {R}^\textbf{N}$, $V(x',x_\textbf{N})=v(x')\otimes \delta (x_{\textbf{N}})$, the estimate, similar to (1.2), for the quantity $\textrm{LT}_{\gamma }(\textbf{H})$ was found, with the integral of $v(x')^{\textbf{N}+2\gamma }$ on the right-hand side.

Quite recently, in [28, 29], an approach was developed to establish eigenvalue estimates and LT type inequalities for singular potentials of the form $\mu =v(x)\varpi$, where $\varpi$ is a fixed measure satisfying the ‘upper Ahlfors condition’,

1.3 $$\begin{aligned} \varpi (B(x,r))\le A r^{s},\quad s>0, \end{aligned}$$

or its two-sided version, and $v(x)\ge 0,$ $x\in \operatorname {supp}\varpi$ is a weight function. Here, *B*(*x*, *r*) is the ball with radius *r* centered at *x*. Note that $s=\textbf{N}$ corresponds to an absolutely continuous measure and thus to the usual Schrödinger operator (1.1). The estimate in [28] has the form

1.4 $$\begin{aligned} \textrm{LT}_\gamma (\textbf{H})\le C \int v(x)^{\theta }\varpi (\textrm{d}x), \qquad \theta = \frac{s+2\gamma }{s+2-\textbf{N}},\quad s>\textbf{N}-2, s>0, \end{aligned}$$

where $\gamma >0$ for $\textbf{N}\ge 2$, $\gamma \ge \frac{1}{2}$ for $\textbf{N}=1$, in particular, reproducing the above result by R. Frank and A. Laptev, where $s=\textbf{N}-1$ (the case $\gamma =0$ and $\textbf{N}\ge 2$ was taken care of previously in [29]).

In particular, in the one-dimensional case, $\textbf{N}=1,$ which we are going to discuss further in the present paper, the conditions in (1.4) allow $\gamma =\frac{1}{2}$ as long as $s>0$. For this special value of the parameter $\gamma ,$ thus, $\theta =1$, $\int v\varpi (dx)=\mu (\mathbb {R}^1),$ estimate (1.4) takes the form

1.5 $$\begin{aligned} \textrm{LT}_{\frac{1}{2}}(\textbf{H})\le C_1\mu (\mathbb {R}^1). \end{aligned}$$

This aesthetic estimate, in which the measure $\mu$ itself enters (and not its density), creates an impression that, hopefully, (1.5) is valid for $s=0,$ this means, for an arbitrary non-negative measure $\mu$, not necessarily an absolutely continuous, as well. The proof in [28] breaks down for $s=0$, however, fortunately, this case was taken care of previously, in [17], moreover, with sharp constant $C_1=\frac{1}{2}$ in (1.5). For other values of $\theta ,$ $\theta \ne \frac{1}{2}$, estimate (1.5) does not admit a natural generalization; moreover, it is even hard to guess proper terms.

Generally, the spectra of Schrödinger (Sturm–Liouville) operators with singular potential were studied for a long time, especially, in cases where the potential is a purely discrete measure. Such operators appear in various mathematical models, interesting by themselves but also as an approximation to ‘continuous’ models, and they are still an important field of research today. Here, many methods specific to the one-dimensional case can be applied. However, in these studies, the eigenvalue properties are usually expressed rather indirectly, via zeros and poles of some analytic functions generated by the problem or through continued fractions, rather than directly in terms of the characteristics of the potential.

Therefore, we considered it a challenging problem to find estimates for the distribution of negative eigenvalues and for $\textrm{LT}_\gamma (\textbf{H})$ with general, possibly singular and containing a discrete component, measures acting as a potential. The question on a discrete component, to the best of our knowledge, is presented only in dimension 1. In a higher dimension, a complication arises when defining a self-adjoint operator; say with $\delta$-potentials in $\mathbb {R}^{\textbf{N}},$ $\textbf{N}\ge 2$, one needs to choose among possible self-adjoint realizations, and this choice, of course, may influence the spectrum considerably. Formally, in (1.4), discrete measures are excluded by the condition $s>\textbf{N}-2$.

The feature, common for all dimensions, is the following. Usually, eigenvalue estimates, including **LT**-estimates like (1.2) or (1.4), bound the eigenvalues via integral characteristics of the potential *V*; this means, in particular, that the estimates are the same for equimeasurable potentials and, therefore, do not capture the spacial distribution of the potential. Such dependence needs to be reflected by some different form of the right-hand side of the estimates. The natural idea is to use a kind of averaging of the potential in order to obtain sharper eigenvalue estimates. One of the possible choices of averaging, which we use in this paper, is the instrument created long ago by M. Otelbaev for finding eigenvalue estimates for the one-dimensional Schrödinger operator in the case when the potential, although a function, but quite singular, tends to $+\infty$ at infinity, so that the whole spectrum is discrete, see [25, 26]. The idea of M. Otelbaev’s method involves constructing a certain function, the ‘Otelbaev function,’ which a certain variable window average of the singular potential. In this way, Otelbaev’s function reflects both the size of the measure-potential and the spreading of this measure along the real line.

Recently, the two first-named authors of this paper applied this approach by M. Otelbaev to finding sharp eigenvalue estimates for the Sturm–Liouville operator with a measure serving as a potential, again, in the case when the whole spectrum is discrete, see [16, 24].

In the present paper, we study the spectrum in the case of the potential tending to zero at infinity (in some sense). This case is somewhat more delicate than the one above due to the presence of the essential spectrum filling the positive semi-axis.

We start with some general properties of Radon measures on $\mathbb {R}^1$ and discuss criteria for discreteness of the negative spectrum of the Schrödinger operator with such measure as the potential. After this, we introduce a proper version of the Otelbaev function and describe its basic properties, such as its dependence on the averaging parameter $\alpha$ and behavior at infinity. These properties enable us further on to find *two-sided * eigenvalue estimates for the distribution of negative eigenvalues for the potential being a Radon measure. The measure is not necessarily discrete; however, it may be. In particular, LT type estimates are obtained in terms of the Otelbaev function, providing both upper and lower bounds, including direct and reverse **LT** type inequalities. The latter inequalities cover all positive values of $\gamma$ and are sharp in the sense that both upper and lower estimates involve one and the same expression containing the Otelbaev function. For upper estimates we follow initial approach by Weidl [32], where bracketing is used, with the size of partial intervals adapted to the measure $\mu$. Note that reverse **LT** estimates, see [15], Theorem 4.48, hold only for $\gamma \le \frac{1}{2},$ while ours are valid for all values of $\gamma >0$. Note, however, that for $\gamma =\frac{1}{2}$, the result in [31] gives a sharp value of constant in the reverse **LT** estimate. However, the approach in [31] to the lower **LT** estimate does not easily extend to measure-potentials.

Further on, in a number of examples, detailed calculations show effects of various properties of the measure influencing the eigenvalue distribution. We show that our estimates turn out to be sharper than the ones in [32], and we also show that our estimates improve the subcritical ($\gamma <\frac{1}{2}$) ones in [23]. Additionally, in the case when the condition of discreteness of the negative spectrum is not assumed, we find, in terms of the Otelbaev function, two-sided estimates for the lowest point of the spectrum and the lowest point of the essential spectrum of the operator $\textbf{H}.$

In paper [3] and preprint [4], which appeared recently, a different approach to spectral estimates, including **LT** ones, for nonregular potentials is developed, based upon the ‘landscape function.’ Some results, although less explicit, look similar to ours; however, the conditions on the operator are considerably more restrictive, requiring, in particular, the potential to be a function in the Kato class. Finally, we mention the works [4, 12, 31] and references therein for studies on lower bounds.

## Preliminaries

### Radon Measures on $\mathbb {R}^1$

Throughout this paper, $\mathcal {L}(A)$ denotes the Lebesgue measure of the Borel set $A \subset \mathbb {R}$. For any interval *I* in $\mathbb {R}$, the notation |*I*| refers to the length of the interval. For a set $A\subset \mathbb {R}$, we denote its closure as $\overline{A}$. We let $\mathcal {D}(\cdot )$ and $\mathfrak {d}(\cdot )$ denote the domain of an operator and a form, respectively. By $\sigma (\cdot )$ and $\sigma _{ess}(\cdot )$, we denote the spectrum and the essential spectrum of an operator, respectively. By $\delta _x$, we denote the Dirac measure concentrated at $x \in \mathbb {R}$. For $x \in \mathbb {R}$, by [*x*], we mean the largest integer not exceeding *x*. For $x\in \mathbb {R}$, we use the notation

$$\begin{aligned} \Delta _x(d) = \left[ x - \frac{d}{2},x + \frac{d}{2}\right] . \end{aligned}$$

Recall that a (positive) locally finite measure $\mu$ on the Borel-$\sigma$-algebra $\mathcal {B}(\mathbb {R})$ of $\mathbb {R}$ is a *Radon measure* if it satisfies the conditions of local finiteness, outer regularity and inner regularity:

$$\begin{aligned} \mu (K)&< \infty \text { for all } K \subset \mathbb {R} \text { compact,}\\ \mu (K)&= \inf \{ \mu (O); O \supset K, ~ O \text { open}\} \text { for all } K \subset \mathbb {R} \text { compact,}\\ \mu (O)&= \sup \{ \mu (K); K \subset O, ~ K \text { compact}\} \text { for all } O \subset \mathbb {R} \text { open.} \end{aligned}$$

Later, we will use the following simple lemma:

#### Lemma 2.1

Let $\mu$ be a non-negative Radon measure on $\mathbb {R}$ and $x\in \mathbb {R}$. Then, $\mu ([x - \tau ,x)) \rightarrow 0$ as $\tau \rightarrow 0$.

#### Proof

For $\varepsilon >0$, the inner regularity says that

$$\begin{aligned} \mu ((x-2\tau ,x)) = \sup \{ \mu (K); K \subset (x-2\tau ,x), ~ K \text { compact}\}. \end{aligned}$$

Therefore, there exists a compact set $K_\varepsilon \subset (x-2\tau ,x)$ such that $\mu ((x-2\tau ,x)) - \mu (K_\varepsilon ) < \varepsilon$. Since $K_\varepsilon \subset (x-2\tau ,x)$ is compact, it follows for sufficiently small $\tau _\varepsilon$ that $[x-\tau _\varepsilon ,x) \cap K_\varepsilon = \emptyset$. Therefore, $[x-\tau _\varepsilon ,x)\subset (x-2\tau ,x) \setminus K_\varepsilon$, and consequently, $\mu ([x-\tau _\varepsilon ,x))<\varepsilon$. $\square$

### Formulation of the Problem

Let $\mu$ be a non-negative Radon measure on $\mathbb {R}$. We consider the following sesquilinear form

2.1 $$\begin{aligned} \textbf{a}_\mu (f,g)=\int _{\mathbb {R}}f'\overline{g'} ~\textrm{d}x - \int _{\mathbb {R}} f\overline{g}~\textrm{d}\mu \end{aligned}$$

with the domain

$$\begin{aligned} \mathfrak {d}(\textbf{a}_\mu )=\left\{ f\in H^1(\mathbb {R}): \int _{\mathbb {R}} |f|^2 \textrm{d}\mu < \infty \right\} ; \end{aligned}$$

the corresponding quadratic form will be denoted by $\textbf{a}_\mu [f]:=\textbf{a}_\mu (f,f)$. We recall the Brinck condition for $\mu$, see [10]: There exists $C>0$ such that

2.2 $$\begin{aligned} \mu ([x,x+1]) < C \quad \text {for any } x\in \mathbb {R}. \end{aligned}$$

It is known that (2.2) guarantees that the form $\textbf{a}_\mu$ is lower semi-bounded and closed; see [16] or [22]. Since $\mu$ is sign-definite, the converse is also true:

#### Lemma 2.2

Let $\mu$ be a non-negative Radon measure. The following statements are equivalent:

1. The Brinck condition (2.2) holds.
2. The sesquilinear form $\textbf{a}_\mu$ is lower semi-bounded and closed.

#### Proof

Due to [16, Prop. II.4] or [22], it remains to show that (2) implies (1). Assuming that (1) does not hold, for any fixed $C>0$, there exists $x\in \mathbb {R}$, say, $x=\frac{1}{2}$, such that $\mu ([x,x+1])\ge 3C + \pi ^2$. We define

$$\begin{aligned} u(x):= {\left\{ \begin{array}{ll} 1-\cos \left( \pi \left( x-2\right) \right) , ~& x \in [0,2],\\ 0, &  x \not \in [0,2]. \end{array}\right. } \end{aligned}$$

Then, $\Vert u\Vert ^2 = 3$, while $u \ge 1$ on $[\frac{1}{2}, \frac{3}{2}]$. Therefore,

$$\begin{aligned} \textbf{a}_\mu [u] = \pi ^2 - \int _{0}^2 u^2\textrm{d}\mu \le \pi ^2 - \mu \left( \left[ \frac{1}{2} , \frac{3}{2} \right] \right) . \end{aligned}$$

Hence,

$$\begin{aligned} \textbf{a}_\mu [u]\le \left( \frac{\pi ^2}{3} - \frac{\mu ([1/2,3/2])}{3} \right) \Vert u\Vert ^2 \le -C\Vert u\Vert ^2. \end{aligned}$$

Since $C>0$ was arbitrary, we conclude that $\textbf{a}_\mu$ is not semi-bounded. $\square$

From now on, we will always assume that $\mu$ satisfies (2.2), so that $\textbf{a}_\mu$ is semi-bounded, closed, symmetric, and densely defined. We investigate the spectral properties of the operator $\textbf{H}_\mu$ associated with $\textbf{a}_\mu$ in the sense of Kato’s first representation theorem [18, Theorem 2.6].

### Discreteness of the Negative Spectrum of $\textbf{H}_{\mu }$

Spectral properties of the operator $\textbf{H}_\mu$ for an absolutely continuous measure $\mu$ are well known, see, e.g., [6]. For our more general case, we refer to [21]. There, the measure $\mu$ is even allowed to be non-sign-definite.

We recall the general abstract criterion for discreteness of the negative spectrum of Schrödinger-like operators; see Theorem 1.3 in [6]. Being applied to the operator $\textbf{H}_{\epsilon ,\mu }$ defined by the lower semi-bounded quadratic form

$$\begin{aligned} \textbf{h}_{\epsilon ,\mu }[u]=\textbf{a}[u]-\epsilon \pmb {\mu }[u], \ \textbf{a}[u]=\int _{\mathbb {R}^1}|u'(x)|^2dx, \ \pmb {\mu }[u]=\int _{\mathbb {R}^1} |u(x)|^2 \textrm{d}\mu (x), \end{aligned}$$

on its natural domain, this theorem establishes that the negative spectrum of $\textbf{H}_{\epsilon ,\mu }$ is discrete for all $\epsilon >0$ if and only if the quadratic form $\pmb {\mu }[u]$ is compact in the Sobolev space $H^1(\mathbb {R}^1)$.

The necessary and sufficient condition of this compactness is contained in Theorem 2.5 in [21]:

#### Theorem 2.3

The quadratic form $\pmb {\mu }$ is compact in the Sobolev space $H^1(\mathbb {R}^1)$ if and only if, for any fixed $a>0$, the functions

$$\begin{aligned} G_+(t)=e^t\int _t^{\infty }e^{-s}\textrm{d}\mu (s), \, G_{-}(t)=e^{-t}\int _{-\infty }^t e^{s}\textrm{d}\mu (s) \end{aligned}$$

satisfy

$$\begin{aligned} \lim _{x\rightarrow \pm \infty }\int _{x-a}^{x+a}G_{\pm }(t)^2 \textrm{d}t=0. \end{aligned}$$

#### Remark 2.4

For a positive measure, this condition is equivalent to

2.3 $$\begin{aligned} \mu ((x-a,x+a))\rightarrow 0 \quad \text{ as } \, x\rightarrow \pm \infty \end{aligned}$$

for some, and therefore, for every $a>0.$

We will always suppose that these conditions are fulfilled.

#### Remark 2.5

Theorem 2.3 is valid for a non-sign-definite measure $\mu$ as well. Finding eigenvalue estimates for such measures, even for absolutely continuous ones, while one needs to trace the possible cancelation of the contributions of the positive and negative components, is a hard and challenging problem. See [11] for some partial results.

## Otelbaev’s Function

In this section, we introduce Otelbaev’s function and establish its properties. This function, introduced in [25] for the study of Schrödinger operators on $\mathbb {R}^1$ having purely discrete spectrum, is a special kind of averaging of the potential. This construction was extended to measure-potentials in [16, 24].

### Definition 3.1

Let $\mu$ be a non-negative Radon measure on $\mathbb {R}$; we fix the averaging parameter $\alpha >0$. Then, we define the Otelbaev function by

3.1 $$\begin{aligned} q^*_\alpha (x) := q_{\alpha , \mu }^*(x) := \inf \left\{ \frac{1}{d^2}: \mu \left( \Delta _x(d)\right) < \frac{1}{\alpha d} \right\} . \end{aligned}$$

We will write $q_{\alpha , \mu }^*(x)$ or $q_\alpha ^*(x)$ if we want to emphasize the dependence on the particular measure $\mu$ or, otherwise, if no confusion between different measures is possible.

Obviously, for any fixed *x* and sufficiently small $d>0$, the inequality in (3.1) holds; for *d* large enough, this inequality does not hold, provided $\mu$ is not the zero measure. By monotonicity in *d*, $q_\alpha ^*$ is well defined. Moreover, we will show that $q_\alpha ^*$ is continuous, and if $\mu (\mathbb {R})>0$, then it is strictly positive. We will also consider the auxiliary quantity

$$\begin{aligned} d_\alpha (x) := \frac{1}{\sqrt{q_\alpha ^*(x)}}. \end{aligned}$$

### Remark 3.2

If $\mu$ is a* continuous* measure, that is, it contains no point masses, then $d_\alpha (x)$ is the unique solution to the equation

3.2 $$\begin{aligned} \mu \left( \Delta _x(d)\right) = \frac{1}{\alpha d}. \end{aligned}$$

If $\mu$ contains point masses, this statement may be wrong. Indeed, take $\mu = \delta _{-1} + \delta _{1}$. Then, for any $0<\tau < 2$ and $\kappa \ge 2$,

$$\begin{aligned} \mu \left( \left[ -\frac{\tau }{2},\frac{\tau }{2}\right] \right) = 0 < \frac{1}{2\tau }, \qquad \mu \left( \left[ -\frac{\kappa }{2},\frac{\kappa }{2}\right] \right) = 2 > \frac{1}{2\kappa }. \end{aligned}$$

Therefore, $q_1^*(0) = 1/4$ and $d_1(0) = 2$. Since

$$\begin{aligned} \mu \left( \left[ -\frac{d_1(0)}{2},\frac{d_1(0)}{2}\right] \right) = \mu ([-1,1]) > 1/2, \end{aligned}$$

we conclude that $d_1(0)$ does not satisfy (3.2) with $x=0$.

In general, $d_\alpha (x)$ is the length of the smallest interval centered at *x* such that its $\mu$-measure is not less than $1/(\alpha d_\alpha (x))$. More formally, this property can be stated as follows:

### Lemma 3.3

Let $\alpha >0$ and $x\in \mathbb {R}$. Then

$$\begin{aligned} \mu \left( \Delta _x(d_\alpha (x)) \right) \ge \frac{1}{\alpha d_\alpha (x)} \end{aligned}$$

and

$$\begin{aligned} \mu \left( \Delta _x(d_\alpha (x) - \varepsilon ) \right) < \frac{1}{\alpha (d_\alpha (x) - \varepsilon )} \end{aligned}$$

for any $$0< \varepsilon < d_\alpha (x)$$.

### Proof

Assume that the first estimate breaks down, that is, $\mu \left( \Delta _x(d_\alpha (x)) \right) < \frac{1}{\alpha d_\alpha (x)}.$ By Lemma 2.1,

$$\begin{aligned} \mu \left( \left[ x - \frac{d_\alpha (x) + \varepsilon }{2}\right. , \left. x - \frac{d_\alpha (x)}{2}\right) \right) \rightarrow 0,\, \mu \left( \left( x + \frac{d_\alpha (x) }{2}\right. , \left. x + \frac{d_\alpha (x)+ \varepsilon }{2}\right] \right) \rightarrow 0, \end{aligned}$$

as $\varepsilon \rightarrow 0$. Therefore, $\mu \left( \Delta _x(d_\alpha (x)+\varepsilon ) \right) \rightarrow \mu \left( \Delta _x(d_\alpha (x)\right)$. Since $\frac{1}{\alpha (d_\alpha (x)+\varepsilon )} \rightarrow \frac{1}{\alpha d_\alpha (x)},$ we obtain, due to our assumption, for sufficiently small $\varepsilon >0$:

$$\begin{aligned} \mu \left( \Delta _x(d_\alpha (x)+\varepsilon ) \right) < \frac{1}{\alpha (d_\alpha (x)+\varepsilon )}. \end{aligned}$$

Hence, by the definition of $q_\alpha ^*$, we obtain $\frac{1}{(d_\alpha (x) + \varepsilon )^2} \ge q_\alpha ^*(x)$, and this contradicts $q_\alpha ^*(x) = 1/ d_\alpha ^2(x)$.

If the second statement of the lemma is wrong, there should exist $\varepsilon \in (0,d_\alpha (x))$ such that

$$\begin{aligned} \mu \left( \Delta _x(d_\alpha (x)-\varepsilon ) \right) \ge \frac{1}{\alpha (d_\alpha (x) - \varepsilon )}. \end{aligned}$$

Then, for any $d\ge d_\alpha (x) - \varepsilon$,

$$\begin{aligned} \mu \left( \Delta _x(d) \right) \ge \mu \left( \Delta _x(d_\alpha (x)-\varepsilon ) \right) \ge \frac{1}{\alpha (d_\alpha (x) -\varepsilon )} \ge \frac{1}{\alpha d}. \end{aligned}$$

Therefore,

$$\begin{aligned} q^*_\alpha (x)&= \inf \left\{ \frac{1}{d^2}: \mu \left( \Delta _x(d)\right)< \frac{1}{\alpha d} \right\} \\&= \inf \left\{ \frac{1}{d^2}: \mu \left( \Delta _x(d)\right)< \frac{1}{\alpha d} \; \text {and} \; d< d_\alpha (x) - \varepsilon \right\} . \end{aligned}$$

In particular,

$$\begin{aligned} q_\alpha ^*(x) \ge \frac{1}{(d_\alpha (x) - \varepsilon )^2}, \end{aligned}$$

and this contradicts $q_\alpha ^*(x) = 1/ d_\alpha ^2(x)$. $\square$

In [16], Otelbaev’s function was defined in a slightly different way, i.e., in the *infimum* defining $q_\alpha ^*$, the condition $\mu (\Delta _x(d)) \le \frac{1}{\alpha d}$ was used instead of the present condition with the strict inequality. We will briefly verify that the definition in [16] and the present one are equivalent; the present one is more convenient.

### Lemma 3.4

The following equalities hold true:

$$\begin{aligned} q^*_\alpha (x) = \inf \left\{ \frac{1}{d^2}: \mu \left( \Delta _x(d)\right) \le \frac{1}{\alpha d} \right\} \end{aligned}$$

and

3.3 $$\begin{aligned} d_\alpha (x) = \sup \left\{ d: \mu \left( \Delta _x(d)\right) \le \frac{1}{\alpha d} \right\} . \end{aligned}$$

### Proof

Due to the relation between $q^*_\alpha$ and $d_\alpha$, we only need to show the second statement. Further, it suffices to prove the statement for $\alpha = 1$; so we denote the right-hand side of (3.3) by $\tilde{d}_1(x)$. For $x\in \mathbb {R}$, obviously, $d_1(x)\le \tilde{d}_1(x)$. To show the converse estimate, we choose any $d>d_1(x)$. Then, by Lemma 3.3,

$$\begin{aligned} \mu \left( \Delta _x(d)\right) \ge \mu \left( \Delta _x(d_1(x))\right) \ge \frac{1}{d_1(x)} > \frac{1}{d} \end{aligned}$$

for any $d>d_1(x)$. Therefore,

$$\begin{aligned} \tilde{d}_1(x)&= \sup \left\{ d> 0: ~\mu \left( \Delta _x(d)\right) < \frac{1}{d}\right\} \\&= \sup \left\{ d > 0: ~\mu \left( \Delta _x(d)\right) \le \frac{1}{d}, \; d\le d_1(x) \right\} . \end{aligned}$$

This implies $\tilde{d}_1(x)\le d_1(x)$. Hence, we conclude that $\tilde{d}_1(x)=d_1(x)$. $\square$

Now, we are ready to derive some crucial properties of the function $q_\alpha ^*$.

### Proposition 3.5

Let $\mu$ be a non-negative Radon measure on $\mathbb {R}$. The following statements hold true:

1. If $\mu \ne 0$, then $q_\alpha ^*(x) > 0$ for every $x \in \mathbb {R}$.
2. $q_\alpha ^*(x)$ is a jointly continuous function of $(\alpha , x) \in (0, \infty ) \times \mathbb {R}$.

### Proof

By the definition, $q_{\alpha , \mu }^*(x) = q_{1, \alpha \mu }^*(x)$. Hence, (1) follows from the statement for $\alpha = 1$, cf. [16, Prop. III.3]. To verify (2), note that whenever there is a sequence $(\alpha _n, t_n)_{n \in \mathbb {N}}$ such that $(\alpha _n, t_n) \rightarrow (\alpha , t)$, then $\alpha _n \mu (\cdot - t_n) \rightarrow \alpha \mu (\cdot - t)$ in weak$^*$ topology, i.e., for any $f \in C_c(\mathbb {R}),$ by the dominated convergence theorem,

$$\begin{aligned} \int&f(x) ~d(\alpha _n \mu (\cdot - t_n))(x) = \alpha _n \int f(x + t_n)~ \textrm{d}\mu (x) \\&\overset{n \rightarrow \infty }{\longrightarrow }\ \alpha \int f(x + t) ~\textrm{d}\mu (x) = \int f(x) ~d(\alpha \mu (\cdot - t))(x). \end{aligned}$$

Hence, by [16, Prop. III.5], for each $x \in \mathbb {R}$,

$$\begin{aligned} q_{\alpha _n, \mu }^*(x - t_n) = q_{1, \alpha _n \mu (\cdot - t_n)}^*(x) \longrightarrow q_{1, \alpha \mu (\cdot - t)}^*(x) = q_{\alpha , \mu }^*(x-t), \end{aligned}$$

proving that $q_{\alpha , \mu }^*(x)$ is jointly continuous in $\alpha$ and *x*. $\square$

We will now show that Otelbaev’s function has a controlled variation.

### Lemma 3.6

Let $\mu$ be a non-negative Radon measure on $\mathbb {R}$. Let $\alpha >0$ and $x\in \mathbb {R}$. Then, for all points $y \in \Delta _x(d_\alpha (x))$ and $z \in \Delta _x\left( d_\alpha (x)/2\right)$, the following inequalities hold true:

$$\begin{aligned} \frac{1}{4}q^*_\alpha (z) \le q^*_\alpha (x) \le 4q^*_\alpha (y). \end{aligned}$$

### Proof

First, we prove the estimate on the right-hand side. Let $y \in \Delta _x(d_\alpha (x))$. Then, $\Delta _x(d_\alpha (x)) \subset \Delta _y(2d_\alpha (x))$. Hence, by Lemma 3.3,

$$\begin{aligned} \mu \left( \Delta _y(2d_\alpha (x))\right) \ge \mu \left( \Delta _x(d_\alpha (x))\right) \ge \frac{1}{\alpha d_\alpha (x)} \ge \frac{1}{2\alpha d_\alpha (x)}. \end{aligned}$$

Therefore, by the definition of $q_\alpha ^*$,

$$\begin{aligned} q_\alpha ^*(y) \ge \frac{1}{4d_\alpha ^2(x)} = \frac{1}{4}q^*_\alpha (x). \end{aligned}$$

To show the estimate on the left-hand side, we let $z \in \Delta _x\left( d_\alpha (x)/2\right)$. For sufficiently small $\varepsilon >0$, the following inclusion holds true:

$$\begin{aligned} \Delta _z\left( \frac{d_\alpha (x) - 2\varepsilon }{2}\right) \subset \Delta _x(d_\alpha (x) - \varepsilon ). \end{aligned}$$

Therefore, by Lemma 3.3,

$$\begin{aligned} \mu \left( \Delta _z\left( \frac{d_\alpha (x) - 2\varepsilon }{2}\right) \right) \le \mu \left( \Delta _x(d_\alpha (x) - \varepsilon ) \right)< \frac{1}{\alpha (d_\alpha (x) - \varepsilon )} < \frac{1}{\alpha \left( \frac{d_\alpha (x) - 2\varepsilon }{2}\right) }. \end{aligned}$$

Since $q_\alpha ^*(z) \le \frac{4}{(d_\alpha (x) - 2\varepsilon )^2}$ for all sufficiently small $\varepsilon >0$, by the definition of $q_\alpha ^*$, we arrive at $q_\alpha ^*(z) \le \frac{4}{d_\alpha ^2(x)} = 4q_\alpha ^*(x)$, just what we need. $\square$

Next, we give a reformulation of the Brinck condition (2.2) in terms of $q_\alpha ^*$:

### Lemma 3.7

Let $\mu$ be a non-negative Radon measure. The following statements are equivalent:

1. For any $\alpha >0$, $q^*_\alpha$ is a bounded function.
2. The condition (2.2) is satisfied.

### Proof

If (1) does not hold, there exists a sequence $\{x_k\}$ such that $q_\alpha ^*(x_k)>k$, or equivalently, $d_\alpha (x_k)< 1/\sqrt{k}$. By Lemma 3.3,

$$\begin{aligned} \mu \left( \Delta _{x_k}(1)\right) \ge \mu \left( \Delta _{x_k}(d_\alpha (x_k))\right)> \frac{1}{\alpha d_\alpha (x_k)} > \frac{\sqrt{k}}{\alpha }, \end{aligned}$$

and hence, (2) is violated.

On the opposite, if (1) holds, so that $q_\alpha ^*(x)<C$ for all $x\in \mathbb {R}$, it follows, $d_\alpha (x) > 1/\sqrt{C}$, and Lemma 3.3 implies

$$\begin{aligned} \mu \left( \Delta _x\left( 1/\sqrt{C}\right) \right) <\frac{\sqrt{C}}{\alpha }. \end{aligned}$$

Therefore,

$$\begin{aligned} \mu ([x,x+1]) < ([\sqrt{C}]+1) \frac{\sqrt{C}}{\alpha }, \end{aligned}$$

which gives (2). $$\square $$

From Lemmas 2.2 and 3.7, we derive the following criterion for semi-boundedness of the quadratic form $\textbf{a}_\mu$ in terms of $q_\alpha ^*$.

### Corollary 3.8

Let $\mu$ be a non-negative Radon measure. Then the sesquilinear form $\textbf{a}_\mu$ is semi-bounded if and only if for any fixed $\alpha >0$, the function $q_\alpha ^*$ is bounded.

Next, we study the behavior of the Otelbaev function at infinity.

### Lemma 3.9

For any $\alpha >0,$ the following statements are equivalent:

1. $$q^*_\alpha (x) \rightarrow 0$$ as $$x\rightarrow \infty $$;
2. For any fixed $d>0$, $\mu \left( \Delta _x(d)\right) \rightarrow 0$ as $x\rightarrow \infty$.

The second property is just a reformulation of (2.3). Therefore, both properties above, by Theorem 2.3, are equivalent to the discreteness of the negative spectrum of the operator $\textbf{H}=-\frac{d^2}{dx^2}-\mu$.

### Proof

Assume that (1) does not hold. Then, $q^*_\alpha (x_k)>C$ for some sequence $\{x_k\}$ tending to infinity and some $C>0$. Therefore, $d_\alpha (x_k)< 1/\sqrt{C}$. Now, by Lemma 3.3,

$$\begin{aligned} \mu \left( \Delta _{x_k}\left( 1/\sqrt{C}\right) \right) \ge \mu \left( \Delta _{x_k}(d_\alpha (x_k))\right) \ge \frac{1}{\alpha d_\alpha (x_k)} \ge \frac{\sqrt{C}}{\alpha }, \end{aligned}$$

which contradicts (2).

Conversely, if (1) holds, but (2) does not, there exists a sequence $\{x_k\}$ tending to infinity and constants *d*, $C>0$ such that

3.4 $$\begin{aligned} \mu \left( \Delta _{x_k}(d)\right) > C. \end{aligned}$$

We choose $\varepsilon >0$ such that

3.5 $$\begin{aligned} \frac{d}{2}< \frac{1}{2\sqrt{\varepsilon }}, \qquad \frac{\sqrt{\varepsilon }}{\alpha } < C. \end{aligned}$$

Since (1) holds, there exists $N>0$ such that $q_\alpha ^*(x)<\varepsilon$ for all $|x| >N$. Therefore, $d_\alpha (x)> 1/\sqrt{\varepsilon }$ for all $|x| >N$. Relation (3.5) and the second part of Theorem 3.3 imply now

$$\begin{aligned} \mu \left( \Delta _{x_k}(d)\right) \le \mu \left( \Delta _{x_k}\left( 1/\sqrt{\varepsilon }\right) \right) \le \frac{\sqrt{\varepsilon }}{\alpha } < C, \end{aligned}$$

for sufficiently large *k*, such that $|x_k| > N$. This contradicts (3.4). $\square$

For different values of the averaging parameter, Otelbaev’s functions are equivalent:

### Lemma 3.10

Let $\mu$ be a non-negative Radon measure. If $0<\beta <\alpha$, then

$$\begin{aligned} q_\beta ^*(x) \le q_\alpha ^*(x) \le \frac{\alpha ^2}{\beta ^2} q_\beta ^*(x), \end{aligned}$$

for all $$x\in \mathbb {R}$$.

### Proof

For a fixed $x\in \mathbb {R}$, it is clear that $\alpha \mapsto q_\alpha ^*(x)$ is an increasing function, so that $q_\beta ^*(x) \le q_\alpha ^*(x)$.

Let us show the second inequality. If $q_\beta ^*(x) = q_\alpha ^*(x)$, the statement is obviously true. Otherwise, if $q_\beta ^*(x) < q_\alpha ^*(x)$, then $d_\alpha (x) < d_\beta (x)$; this means that $d_\alpha (x) < d_\beta (x) -\varepsilon$ for some sufficiently small $\varepsilon >0$. Therefore, by Lemma 3.3,

$$\begin{aligned} \frac{1}{\alpha d_\alpha (x)} \le \mu (\Delta _x(d_\alpha (x))) \le \mu (\Delta _x(d_\beta (x) - \varepsilon )) \le \frac{1}{\beta (d_\beta (x) - \varepsilon )}, \end{aligned}$$

for any sufficiently small $\varepsilon >0$. Thus,

$$\begin{aligned} \frac{1}{ d_\alpha (x)} \le \frac{\alpha }{\beta d_\beta (x)}. \end{aligned}$$

Recalling the relation between $q_\alpha ^*$ and $d_\alpha$ completes the proof. $\square$

The following lemma concerns the dependence of $q_\alpha ^*$ on the choice of the measure.

### Lemma 3.11

Let $\mu _1$ and $\mu _2$ be non-negative Radon measures and $\mu =\mu _1+\mu _2$. Then, for any $\alpha >0$,

3.6 $$\begin{aligned} q_{\alpha ,\mu }^*(x) \le 2 \left( q_{\alpha ,\mu _1}^*(x) + q_{\alpha ,\mu _2}^*(x)\right) . \end{aligned}$$

### Proof

By Lemma 3.3,

3.7 $$\begin{aligned} \mu _j(\Delta _x(d_{\alpha ,\mu _j}(x))) \ge \frac{1}{\alpha d_{\alpha ,\mu _j}(x)}, \quad \text {for } x\in \mathbb {R}, \; j=1,2. \end{aligned}$$

We note that

3.8 $$\begin{aligned} d_{\alpha ,\mu _j}(x) \ge d_{\alpha ,\mu }(x) \quad \text {for } j=1,2. \end{aligned}$$

If $d_{\alpha ,\mu _1}(x) = d_{\alpha ,\mu }(x)$, then $q_{\alpha ,\mu _1}^*(x) = q_{\alpha ,\mu }^*(x)$ and (3.6) holds. If the estimate in (3.8) is strict for both $j=1,2$, i.e., for some $\varepsilon >0$,

$$\begin{aligned} d_{\alpha ,\mu _j}(x) - \varepsilon > d_{\alpha ,\mu }(x), \end{aligned}$$

then, by Lemma 3.3,

$$\begin{aligned}  &   \frac{1}{\alpha (d_{\alpha ,\mu _1}(x) - \varepsilon )} + \frac{1}{\alpha (d_{\alpha ,\mu _2}(x) - \varepsilon )} > \mu _1(\Delta _x(d_{\alpha ,\mu _1}(x) - \varepsilon )) + \mu _2(\Delta _x(d_{\alpha ,\mu _2}(x) - \varepsilon ))\\  &   \qquad \ge \mu _1(\Delta _x(d_{\alpha ,\mu }(x))) + \mu _2(\Delta _x(d_{\alpha ,\mu }(x))) = \mu (\Delta _x(d_{\alpha ,\mu }(x))) \ge \frac{1}{\alpha d_{\alpha ,\mu }(x) } \end{aligned}$$

holds true for any sufficiently small $\varepsilon >0$. Now (3.6) follows from

$$\begin{aligned} \frac{1}{\alpha (d_{\alpha ,\mu _1}(x) )} + \frac{1}{\alpha (d_{\alpha ,\mu _2}(x) )} \ge \frac{1}{\alpha d_{\alpha ,\mu }(x) }. \end{aligned}$$

$$\square $$

We conclude this section by showing that Otelbaev’s function satisfies a certain lower bound for its decay at infinity:

### Proposition 3.12

Let $0 \ne \mu$ be a non-negative Radon measure. Then,

$$\begin{aligned} \liminf _{|x| \rightarrow \infty } q_\alpha ^*(x) |x|^2 \ge \frac{1}{4}. \end{aligned}$$

### Proof

We first assume that $\mu$ is supported in some interval $[-R, R]$. Further, we only consider $x > 0$, since the case $x < 0$ can be dealt with analogously.

Since $\mu$ is a compactly supported Radon measure, it is finite. Assume that *x* is large enough such that $2(x+R) > \frac{\mu (\mathbb {R})}{\alpha }$. Then, with $d = 2(x+R)$, we obtain

$$\begin{aligned} \mu (\Delta _x(d)) = \mu ([-R, 2x+R]) = \mu (\mathbb {R}) > \frac{1}{2\alpha (x+R)} = \frac{1}{\alpha d}. \end{aligned}$$

Hence, we see that for every *x* sufficiently large,

$$\begin{aligned} q_\alpha ^*(x) \ge \frac{1}{d^2} = \frac{1}{4(x+R)^2}, \end{aligned}$$

which implies

$$\begin{aligned} \liminf _{x \rightarrow \infty } q_\alpha ^*(x) \cdot x^2 \ge \liminf _{x \rightarrow \infty } \frac{x^2}{4(x+R)^2} = \frac{1}{4}. \end{aligned}$$

If $\mu$ is not compactly supported, then for arbitrary $R > 0$ we have $\mu \ge \textbf{1}_{[-R, R]} \mu$, so

$$\begin{aligned} q_{\mu , \alpha }^*(x) \ge q_{\textbf{1}_{[-R, R]}\mu , \alpha }^*(x), \end{aligned}$$

and therefore

$$\begin{aligned} \liminf _{x \rightarrow \infty } q_{\mu , \alpha }^*(x) \cdot x^2 \ge q_{\textbf{1}_{[-R, R]}\mu , \alpha }^*(x) \cdot x^2 \ge \frac{1}{4}. \end{aligned}$$

The estimate at $-\infty$ is dealt with analogously. This finishes the proof. $\square$

### Remark 3.13

If $\mu$ is compactly supported, then we also have

$$\begin{aligned} \limsup _{|x|\rightarrow \infty } q_\alpha ^*(x) \cdot x^2 \le 1, \end{aligned}$$

which will be clear from the discussion in Example B.3.

## Eigenvalue Estimates

Lemma 3.9 gave us a condition of discreteness of the negative spectrum in terms of the function $q_\alpha ^*$. In terms of the same function, we now find two-sided estimates for the spectral distribution function $N(-\lambda , \textbf{H}_\mu )$ for $\lambda >0$, defined as the number of eigenvalues less than $-\lambda$. The reasoning uses the standard bracketing idea, in the realization of, e.g., [32], adapted to general measures. Further, we always assume that $\mu$ is a non-negative Radon measure that satisfies Brinck’s condition (2.2).

### Auxiliary Lemmas

We consider a covering of $\mathbb {R}$ by closed intervals which have common endpoints. If such endpoint coincides with the position of a point mass of the measure $\mu$ we split this point mass in two, in a special way, and assign each summand to the corresponding interval. Note that besides depending on $\mu$, the construction also depends on $\alpha > 0$.

We start with $a_0 = 0$ and $\gamma _0 = 0$. If $\mu ([a_0,+\infty )) = 0$, then we define $a_{1}= +\infty$. Otherwise, we set

$$\begin{aligned} a_{1} := \sup \left\{ x\in (a_0,+\infty ): \ \mu ([a_0, x)) \le \frac{1}{\alpha |x - a_0|} \right\} . \end{aligned}$$

Since

$$\begin{aligned} \mu ([a_0, x)) \uparrow \mu ([a_0, \infty )) > 0 \quad \text { and } \quad \frac{1}{\alpha |x-a_0|} \downarrow 0 \quad \text {as } x \rightarrow \infty , \end{aligned}$$

$a_1$ is well defined and finite. Further, since both $\mu ([a_0, x)) \rightarrow \mu (\{ a_0\}) < \infty$ and $(\alpha |x-a_0|)^{-1} \rightarrow \infty$ continuously as $x \rightarrow 0$, we also obtain $a_1 > a_0$.

Having constructed $a_1$ in this way, we have the following fact:

#### Lemma 4.1

If $$a_1 < \infty $$, then $$\mu ([a_0, a_1]) \ge \frac{1}{\alpha (a_1 - a_0)}$$.

#### Proof

Assume it was $\mu ([a_0, a_1]) < \frac{1}{\alpha (a_1 - a_0)}$. Since

$$\begin{aligned} \mu ([a_0, a_1 + \varepsilon )) \overset{\varepsilon \rightarrow 0}{\longrightarrow }\ \mu ([a_0, a_1]), \end{aligned}$$

we would have for some $\varepsilon ' > 0$ sufficiently small:

$$\begin{aligned} \mu ([a_0, a_1 + \varepsilon ')) \le \frac{1}{\alpha (a_1 + \varepsilon ' - a_0)}. \end{aligned}$$

But this contradicts the definition of $a_1$. $\square$

If $a_1 = \infty$, we set $I_0 = [a_0, \infty )$, define the measure $\mu _0$ on $I_0$ as the zero measure and end the construction here. If $a_1 < \infty$, we can set (based on the previous lemma) $\gamma _1 = \frac{1}{\alpha (a_1 - a_0)} - \mu ([a_0, a_1)) \ge 0$. We define the interval $I_0$ as $I_0 = [a_0, a_1]$ and the measure $\mu _0$ on $I_0$ as $\mu _0 = \mu \cdot \textbf{1}_{[a_0, a_1)} + \gamma _1 \delta _{a_1}$. We now continue the construction inductively. After having constructed *finitely* many points $a_0,\ldots ,a_k$, intervals $I_0, \ldots , I_{k-1}$, constants $\gamma _1, \ldots , \gamma _k$ and measures $\mu _0, \ldots , \mu _{k-1}$, we define $a_{k+1}$ analogously, that is: if $\mu ([a_k,+\infty )) - \gamma _k= 0$ we set $a_{k+1}= +\infty$ and $I_k = [a_k, \infty )$, $\mu _k = 0$ on $I_k$. Otherwise

$$\begin{aligned} a_{k+1} := \sup \left\{ x\in (a_k,+\infty ): \ \mu ([a_k, x)) - \gamma _k \le \frac{1}{\alpha |x - a_k|} \right\} . \end{aligned}$$

As above, $a_{k+1}$ is well defined. If $a_{k+1} = \infty$, we let $I_k = [a_k, \infty )$, $\mu _k = 0$ on $I_k$ and end the construction here. Otherwise, analogously to Lemma 4.1, we can set $\gamma _{k+1} = \frac{1}{\alpha (a_{k+1} - a_k)} - \mu ([a_k, a_{k+1})) + \gamma _k \ge 0$, $I_k = [a_k, a_{k+1}]$, $\mu _k$ is the measure on $I_k$ given by $\mu _k = \mu \cdot \textbf{1}_{[a_k, a_{k+1})} - \gamma _k \delta _{a_k} + \gamma _{k+1} \delta _{a_{k+1}}$.

For $k \le 0$, we repeat the construction symmetrically, i.e., we construct the points

$$\begin{aligned} a_{k-1} := \inf \left\{ x \in (-\infty , a_k): ~\mu ((x, a_k]) - \gamma _k \le \frac{1}{\alpha (a_k - x)} \right\} \end{aligned}$$

and for $a_{k-1} > -\infty$ we set $I_{k-1} = [a_{k-1}, a_k]$, $\gamma _{k-1} = \frac{1}{\alpha (a_k - x)} - \mu ((a_{k-1}, a_k]) + \gamma _k$. We denote by $\mathbb {K}\subset \mathbb {Z}$ the set of ordinals *k* of these points (with exception of $+\infty$ if such point is present). If $a_k \ne -\infty$ and $a_{k+1} \ne \infty$, we denote by $x_k$ the mid-point of the interval $I_k$. With these definitions, the intervals $I_k$ form a partition of $\mathbb {R}$. We emphasize that this whole construction implicitly depends on the choice of the measure $\mu$ and the constant $\alpha > 0$, even though we do not capture this in the notation of the intervals and the points.

#### Remark 4.2

We want to emphasize that the assumption of Brinck’s condition (2.2) on $\mu$ enforces that $a_k$ cannot converge to some finite point as $k \rightarrow \pm \infty$, i.e., the intervals $I_k$ indeed form a covering of $\mathbb {R}$.

We have now constructed a covering $\{ I_k\}_{k \in \mathbb {K}}$ of $\mathbb {R}$ and a collection of measures $\mu _k$ such that for every Borel set $A \subset \mathbb {R}$: $\mu (A) = \sum _{k \in \mathbb {K}} \mu _k(A_k \cap I_k)$. With this data, we will perform our Neumann bracketing later.

#### Lemma 4.3

The decomposition has the following properties:

1. For $$k \in \mathbb {K}$$ with $$\mu _k(I_k) > 0$$: $$\mu _k(I_k) |I_k| = \frac{1}{\alpha }$$.
2. It is: $$d_\alpha (x_k) = |I_k|$$.

#### Proof

The first part is satisfied by construction. To show the second part, we assume that $|I_k| < d_\alpha (x_k)$, i.e., $a_{k+1} - a_k < d_\alpha (x_k)$. Then,

$$\begin{aligned} x_k - \frac{d_\alpha (x_k) - \varepsilon }{2}< a_k< a_{k+1} < x_k + \frac{d_\alpha (x_k) - \varepsilon }{2} \end{aligned}$$

for all sufficiently small $\varepsilon > 0$. By construction of $a_{k+1}$, we have

$$\begin{aligned} \mu \left( \left[ a_k, x_k + \frac{d_\alpha (x_k) - \varepsilon }{2} \right) \right) > \frac{1}{\alpha \left( x_k + \frac{d_\alpha (x_k) - \varepsilon }{2} - a_k\right) } + \gamma _k \ge \frac{1}{\alpha \left( x_k + \frac{d_\alpha (x_k) - \varepsilon }{2} - a_k \right) }. \end{aligned}$$

On the other hand, by Lemma 3.3,

$$\begin{aligned} \mu \left( \left[ a_k,x_k + \frac{d_\alpha (x_k) - \varepsilon }{2}\right) \right) \le \mu \left( \Delta _{x_k}(d_\alpha (x_k) - \varepsilon ) \right) < \frac{1}{\alpha (d_\alpha (x_k) - \varepsilon )}. \end{aligned}$$

Therefore,

$$\begin{aligned} \frac{1}{\alpha \left( x_k + \frac{d_\alpha (x_k) - \varepsilon }{2} - a_k\right) } < \frac{1}{\alpha (d_\alpha (x_k) - \varepsilon )} \end{aligned}$$

for sufficiently small $\varepsilon > 0$. This is equivalent to

$$\begin{aligned} d_\alpha (x_k) - \varepsilon&< x_k + \frac{d_\alpha (x_k) - \varepsilon }{2} - a_k \\&= \frac{a_{k+1} + a_k}{2} + \frac{d_\alpha (x_k) - \varepsilon }{2} - a_k =\frac{a_{k+1} - a_k}{2} + \frac{d_\alpha (x_k) - \varepsilon }{2}, \end{aligned}$$

and this contradicts our initial assumption that $d_\alpha (x_k) > a_{k+1} - a_k$.

Now, assume that $d_\alpha (x_k) < |I_k|$. Then,

$$\begin{aligned} \Delta _{x_k}(d_\alpha (x_k)) \subset I_k^{int}, \end{aligned}$$

where $I_k^{int}$ is the interior of $I_k$. This and Lemma 3.3 imply

$$\begin{aligned} \mu _k(I_k) \ge \mu (I_k^{int}) \ge \mu (\Delta _{x_k}(d_\alpha (x_k)))> \frac{1}{\alpha d_\alpha (x_k)} > \frac{1}{\alpha |I_k|}. \end{aligned}$$

This contradicts part (1) of the present lemma. $\square$

### Neumann Bracketing

We consider here Sturm–Liouville operators with Neumann boundary conditions on each interval $I_k$, with the potentials being the measure $\mu _k$. To define these operators precisely, we need the following lemmas.

#### Lemma 4.4

[10, Lemma 2]. Let $I \subset \mathbb {R}$ be an interval of length *d* and $f \in H^1(I)$. Then, for all $y \in I$,

$$\begin{aligned} \frac{1}{2d} \int _{I}|f(x)|^2 \textrm{d}x - \frac{d}{2}\int _{I}|f'(x)|^2 dx \le |f(y)|^2 \le \frac{2}{d} \int _{I}|f(x)|^2 \textrm{d}x + d \int _{I}|f'(x)|^2 \textrm{d}x. \end{aligned}$$

#### Lemma 4.5

Let $\alpha >0$ and $I_k$ be some interval as defined in Sect. 4.1. Then, the sesquilinear form

4.1 $$\begin{aligned} \textbf{a}_\mu ^k[f]=\int _{I_k}|f'|^2 \textrm{d}x - \int _{I_k}|f|^2 \textrm{d}\mu _k, \qquad \mathfrak {d}(\textbf{a}_\mu ^k)= H^1(I_k), \end{aligned}$$

is lower semi-bounded, closed, symmetric, and densely defined in $L^2(I_k)$.

#### Proof

If $\mu _k(I_k) = 0$, nothing needs to be proven. Otherwise, it is clear that $\textbf{a}_\mu ^k$ is symmetric and densely defined. Due to Lemma 4.3, $|I_k| \mu _k(I_k) = \frac{1}{\alpha }$. Hence, by Lemma 4.4, for $f\in H^1(I_k)$ we obtain:

$$\begin{aligned} \left| \int _{I_k} |f|^2\textrm{d}\mu _k\right|&\le \frac{2\mu _k(I_k)}{|I_k|} \Vert f\Vert _{L^2(I_k)}^2 + |I_k|\mu _k(I_k) \Vert f'\Vert _{L^2(I_k)}^2\\&\le \frac{2\mu _k(I_k)}{|I_k|} \Vert f\Vert ^2_{L^2(I_k)} + \frac{1}{\alpha + 1} \cdot (1 + \frac{1}{\alpha }) \Vert f'\Vert _{L^2(I_k)}^2. \end{aligned}$$

Since $(1+\alpha ^{-1})\Vert f'\Vert _{L^2(I_k)}^2$ is a non-negative, closed, symmetric and densely defined quadratic form on the domain $H^1(I_k)$ and $\frac{1}{1+\alpha } < 1$, the KLMN-Theorem implies that $\textbf{a}_\mu ^k$ is closed and semi-bounded; see, e.g., [27, Theorem X.17]. $\square$

Our upper estimates for eigenvalues will be, as this is often done, by means of the Neumann bracketing. The standard application of the variational principle (say, the Glazman lemma) is the following.

#### Lemma 4.6

Let $\mu$ be a Radon measure satisfying (2.2) and $\textbf{H}_\mu$ be the operator generated by the sesquilinear form (2.1). Let $\alpha >0$ and $\{I_k\}_{k\in \mathbb {K}}$ be the intervals introduced above. Consider the operators $\textbf{H}_\mu ^k$ associated with the sesquilinear forms (4.1). Then, for every $\lambda >0$, the following inequality holds true:

$$\begin{aligned} N(-\lambda ,\textbf{H}_\mu )\le \sum _{k \in \mathbb {K}} N(-\lambda ,\textbf{H}_\mu ^k). \end{aligned}$$

In our first estimate, we use the intervals $I_k$ corresponding to the special value $\alpha =2$.

#### Lemma 4.7

Under the conditions above, for $\alpha = 2$, the following estimate holds for all $\lambda >0$:

$$\begin{aligned} N(-\lambda , \textbf{H}_\mu ) \le \sum _{k \in \mathbb {K}: ~-\lambda \ge -\frac{1}{|I_k|^2}} 1 = \#\{ k\in \mathbb {K}: \lambda \le 1 /|I_k|^2\}. \end{aligned}$$

#### Proof

Let $\lambda >0$. Let $\textbf{H}_\mu ^k$ be the operators from Lemma 4.6 and $\textbf{a}_\mu ^k$ be the corresponding quadratic forms. Then,

4.2 $$\begin{aligned} N(-\lambda , \textbf{H}_\mu ) \le \sum _{k\in \mathbb {K}} N(-\lambda , \textbf{H}_\mu ^k). \end{aligned}$$

By construction, if some $I_k$ is unbounded, then it follows that $\mu _k(I_k) = 0$, and hence, $N(-\lambda ,H_\mu ^k) = 0$. Therefore, from now on, we will only consider bounded intervals $I_k$. By Lemma 4.4,

$$\begin{aligned} \textbf{a}_\mu ^k[f] \ge \left( 1 - |I_k|\mu _k(I_k) \right) \Vert f'\Vert _{L^2(I_k)}^2 - \frac{2}{|I_k|} \mu _k(I_k) \Vert f\Vert _{L^2(I_k)}^2. \end{aligned}$$

From Lemma 4.3, we know that $2\mu (I_k) = 1/|I_k|$. Hence, the estimate above gives

4.3 $$\begin{aligned} \textbf{a}_\mu ^k[f] \ge \textbf{b}^k[f], \end{aligned}$$

where $\textbf{b}^k$ is the sesquilinear form defined as follows:

$$\begin{aligned} \textbf{b}^k[f] = \frac{1}{2} \Vert f'\Vert _{L^2(I_k)}^2 - \frac{1}{|I_k|^2} \Vert f\Vert _{L^2(I_k)}^2, \qquad \mathfrak {d}(\textbf{b}^k) = H^1(I_k), \, f\in H^1(I_k). \end{aligned}$$

Therefore,

4.4 $$\begin{aligned} \textbf{H}_\mu ^k\ge \textbf{B}^k, \end{aligned}$$

in the sense of quadratic forms, where $\textbf{B}^k$ is the operator associated with $\textbf{b}^k$. Note that $\textbf{B}^k$ is, up to a constant factor, $(-\frac{d^2}{dx^2})_{\mathcal {N}} - c I$, with $(-\frac{d^2}{dx^2})_\mathcal {N}$ being the Neumann Laplacian of the interval $I_k$. Hence, the spectrum of $\textbf{B}^k$ is well known: it is purely discrete and consists of the eigenvalues

$$\begin{aligned} \eta _m = \frac{1}{2}\left( \frac{\pi m}{|I_k|}\right) ^2 - \frac{1}{|I_k|^2},\quad m\in \mathbb {N} \cup \{0\}. \end{aligned}$$

For the lowest eigenvalue of $\textbf{B}^k$, we have $\eta _0 = -1/|I_k|^2$, but the next eigenvalue is already larger than $-\lambda$:

$$\begin{aligned} \eta _1 = \left( \frac{\pi ^2}{2} - 1\right) \frac{1}{|I_k|^2} > 0. \end{aligned}$$

Therefore,

$$\begin{aligned} N(-\lambda ,\textbf{B}^k) = {\left\{ \begin{array}{ll} 1 &  \text {if } -1/|I_k|^2<-\lambda ,\\ 0 &  \text {otherwise}, \end{array}\right. } \end{aligned}$$

and hence, (4.4) implies that $N(-\lambda ,\textbf{H}_\mu ^k) \le 1$. Moreover, from (4.3), we know that

$$\begin{aligned} N(-\lambda ,\textbf{H}_\mu ^k) = 0, \quad \text {for } -\lambda < -\frac{1}{|I_k|^2}. \end{aligned}$$

This all means that the intervals $I_k$ with $-1/|I_k|^2<-\lambda$ contribute with at most one eigenvalue to the sum (4.2), while the remaining intervals do not make any contribution to this sum at all. $\square$

### Two-Sided Estimate for the Spectral Distribution Function

Now, we are ready to obtain the main results of this section, which are expressed in terms of the measure of sublevel sets for $q_\alpha ^*$:

$$\begin{aligned} M_\alpha (\lambda ) : = \{x\in \mathbb {R}: \; q_\alpha ^*(x) \ge \lambda \}. \end{aligned}$$

#### Theorem 4.8

Let $\mu$ be a non-negative Radon measure satisfying (2.2) and $\textbf{H}_\mu$ be the operator generated by sesquilinear form (2.1). Let $\alpha = \left( \pi ^2 + 3/4\right) ^{-1}$. Then, for $\lambda >0$ the following estimates hold true:

4.5 $$\begin{aligned} \frac{1}{2} \min _{y\in M_\alpha (4\lambda )} \sqrt{q^*_{\alpha }(y)} \mathcal {L} (M_\alpha (4\lambda ))&\le N(-\lambda , \textbf{H}_\mu ) \le \max _{y\in M_2(\lambda /4)} \sqrt{q^*_{2}(y)} \cdot \mathcal {L} (M_2(\lambda /4)),\end{aligned}$$

4.6 $$\begin{aligned} \sqrt{\lambda } \mathcal {L}(M_\alpha (4\lambda ))&\le N(-\lambda , \textbf{H}_\mu ) \le \sqrt{\Vert q_2^*\Vert _{\infty }} \mathcal {L} (M_2(\lambda /4)),\end{aligned}$$

4.7 $$\begin{aligned} \sup _{\sqrt{\eta }\ge \lambda } \left( \eta \cdot \mathcal {L}(M_\alpha (4\eta ))\right)&\le N(-\lambda , \textbf{H}_\mu ) \le 4 \int _{M_2(\lambda /4)} \sqrt{q_2^*(y)}\textrm{d}y. \end{aligned}$$

Here, $\mathcal {L}$ is the Lebesgue measure.

#### Remark 4.9

Theorem 4.8 does not make *a-priori* assumptions about the nature of the negative spectrum of $\textbf{H}_{\mu }$. The inequalities should be understood in the following way. If, for a given $\lambda >0$, one of the quantities on the right-hand side is finite, then the spectrum below $-\lambda$ is finite, and the inequalities hold. On the other hand, if some of the quantities on the left-hand side is infinite, the spectrum of $\textbf{H}_\mu$ in $(-\infty , -\lambda ]$ is infinite and thus contains at least one point of the essential spectrum.

#### Proof

**The upper bound.** We apply Lemma 4.7, which gives

4.8 $$\begin{aligned} N(-\lambda , \textbf{H}_\mu ) \le \sum _{k \in \mathbb {K}:~ -\lambda \ge -\frac{1}{|I_k|^2}} 1, \end{aligned}$$

where the $I_k$ are the intervals defined as Sect. 4.1 with $\alpha = 2$. For those $k\in \mathbb {K}$ for which

4.9 $$\begin{aligned} -\lambda \ge -\frac{1}{|I_k|^2}, \end{aligned}$$

we know from Lemma 4.3 that $I_k = \Delta _{x_k}(d_2(x_k))$. Therefore, for any $y \in I_k$, Lemma 3.6 and (4.9) imply that

$$\begin{aligned} q_2^*(y)\ge \frac{1}{4}q_2^*(x_k) = \frac{1}{4|I_k|^2}\ge \frac{\lambda }{4}. \end{aligned}$$

Hence, we obtain

$$\begin{aligned} 1 = |I_k| \cdot \frac{1}{|I_k|} \le \frac{1}{|I_k|} \cdot \mathcal {L} \left( \left\{ x\in I_k: q_2^*(x)\ge \frac{\lambda }{4} \right\} \right) . \end{aligned}$$

Then,

$$\begin{aligned} N(-\lambda , \textbf{H}_\mu )&\le \sum _{k \in \mathbb {K}:~-\lambda \ge -\frac{1}{|I_k|^2}} \frac{1}{|I_k|} \cdot \mathcal {L} \left( \left\{ x\in I_k: q_2^*(x)\ge \frac{\lambda }{4} \right\} \right) \\&\le \max _{y\in \left\{ x\in I_k: q_2^*(x)\ge \frac{\lambda }{4} \right\} } \sqrt{q_2^*(y)} \cdot \mathcal {L} \left( \left\{ x\in \mathbb {R}: q_2^*(x)\ge \frac{\lambda }{4} \right\} \right) . \end{aligned}$$

This gives the first upper bound. The second upper bound is an obvious consequence of the first one. The third upper bound is obtained from:

$$\begin{aligned}  &   N(-\lambda , \textbf{H}_\mu ) \le \sum _{-\lambda \ge -\frac{1}{|I_k|^2}} \int _{I_k}\frac{1}{|I_k|}\textrm{d}y \\  &   \quad \le 4\sum _{-\lambda \ge -\frac{1}{|I_k|^2}} \int _{I_k}\sqrt{q^*_{\alpha }(y)}\textrm{d}y \le 4 \int _{\left\{ x\in \mathbb {R}: q_2^*(x)\ge \frac{\lambda }{4}\right\} } \sqrt{q_2^*(y)}\textrm{d}y. \end{aligned}$$

**The lower bound.** For a fixed $\lambda >0$, we define intervals

$$\begin{aligned}&J_k : = \left( \frac{k-1}{\sqrt{\lambda }}, \frac{k}{\sqrt{\lambda }}\right) \\&\tilde{J}_k := \left( \frac{k-1}{\sqrt{\lambda }}+ \frac{1}{4\sqrt{\lambda }}, \frac{k}{\sqrt{\lambda }} - \frac{1}{4\sqrt{\lambda }}\right) \\&\tilde{\tilde{J}}_k := \left( \frac{k-1}{\sqrt{\lambda }} + \frac{3}{8\sqrt{\lambda }}, \frac{k}{\sqrt{\lambda }} - \frac{3}{8\sqrt{\lambda }}\right) \end{aligned}$$

for $k\in \mathbb {Z}$. Note that $J_k$, $\tilde{J}_k$, and $\tilde{\tilde{J}}_k$ have the same centerpoint and their lengths, respectively, are $\frac{1}{\sqrt{\lambda }}$, $\quad \frac{1}{2\sqrt{\lambda }}$, and $\frac{1}{4\sqrt{\lambda }}$. We define the orthogonal system of test functions

$$\begin{aligned} f_k(x):= {\left\{ \begin{array}{ll} 1-\cos \left( 2\pi \sqrt{\lambda }\left( x-\frac{k}{\sqrt{\lambda }}\right) \right) , ~& x \in J_k,\\ 0, &  x \not \in J_k, \end{array}\right. } \end{aligned}$$

for $k\in \mathbb {Z}$. Since $f_k\ge 1$ in $\tilde{J}_k$, we can estimate the value of the quadratic form on $f_k$:

$$\begin{aligned} \textbf{a}_\mu [f_k] = 2\pi ^2\sqrt{\lambda } - \int _{J_k} f_k^2~\textrm{d}\mu \le 2\pi ^2\sqrt{\lambda } - \mu (\tilde{J}_k). \end{aligned}$$

We set $\alpha = \left( \pi ^2 + \frac{3}{4}\right) ^{-1}$ and suppose that there exists $\xi \in \tilde{\tilde{J}}_k$ such that $q_{\alpha }^*(\xi ) > 4\lambda$. Then, by the definition of the function $q^*_\alpha$,

$$\begin{aligned} \mu (\tilde{J}_k) \ge \mu \left( \left[ \xi - \frac{1}{4\sqrt{\lambda }},\xi + \frac{1}{4\sqrt{\lambda }} \right] \right) > \frac{\pi ^2 + \frac{3}{4}}{\frac{1}{2\sqrt{\lambda }}} = \left( 2\pi ^2 + \frac{3}{2}\right) \sqrt{\lambda }. \end{aligned}$$

Therefore, $\textbf{a}_\mu [f_k] < -\frac{3}{2}\sqrt{\lambda }$. Since $\Vert f_k\Vert ^2 = \frac{3}{2\sqrt{\lambda }}$, the last estimate gives

$$\begin{aligned} \textbf{a}_\mu ^k[f_k] < - \lambda \Vert f_k\Vert _{L^2(\mathbb {R})}^2. \end{aligned}$$

Now, we conclude that, for $k\in \mathbb {Z}$ such that

$$\begin{aligned} \left\{ x\in \tilde{\tilde{J}}_k+\frac{j}{4\sqrt{\lambda }}: q^*_{\alpha }(x) \ge 4\lambda \right\} \ne \emptyset , \end{aligned}$$

$f_k$ satisfies the last estimate. Moreover, since $\{f_k\}$ have disjoint supports, these functions are linearly independent. Therefore, the variational principle implies

$$\begin{aligned} N(-\lambda ,\textbf{H}_\mu ) \ge \sum _{ k \in \mathbb {Z}:~\left\{ x\in \tilde{\tilde{J}}_k: q^*_{\alpha }(x) \ge 4\lambda \right\} \ne \emptyset } 1 = 4\sqrt{\lambda } \sum _{k \in \mathbb {Z}:~ \left\{ x\in \tilde{\tilde{J}}_k: q^*_{\alpha }(x) \ge 4\lambda \right\} \ne \emptyset } \frac{1}{4\sqrt{\lambda }}. \end{aligned}$$

For any interval $I \subset \mathbb {R}$, we denote by $I+d$ the interval $I\subset \mathbb {R}$ shifted by *d*, that is,

$$\begin{aligned} I+d := \{x+d: x\in I\}. \end{aligned}$$

By the arguments above, we know that

4.10 $$\begin{aligned} N(-\lambda ,\textbf{H}_\mu ) \ge \sum _{k \in \mathbb {Z}:~ \left\{ x\in \tilde{\tilde{J}}_k+\frac{j}{4\sqrt{\lambda }}: q^*_{\alpha }(x) \ge 4\lambda \right\} \ne \emptyset } 1 = 4\sqrt{\lambda } \sum _{k \in \mathbb {Z}:~ \left\{ x\in \tilde{\tilde{J}}_k+\frac{j}{4\sqrt{\lambda }}: q^*_{\alpha }(x) \ge 4\lambda \right\} \ne \emptyset } \frac{1}{4\sqrt{\lambda }} \end{aligned}$$

for $j=0,1,2,3$. The intervals $\tilde{\tilde{J}}_k+\frac{j}{4\sqrt{\lambda }}$ cover $\mathbb {R}^1$ with multiplicity 4, therefore, summing (4.10) over *j*, we obtain

$$\begin{aligned} 4N(-\lambda ,H_\mu ) \ge 4\sqrt{\lambda } \cdot \mathcal {L}\left( \left\{ x\in \mathbb {R}: q^*_{\alpha }(x) \ge 4\lambda \right\} \right) , \end{aligned}$$

and this finishes the proof for the lower bound. $\square$

### Estimates for Eigenvalues

It is convenient to reformulate the preceding results as estimates for the individual negative eigenvalues of $\textbf{H}_{\mu }$.

#### Theorem 4.10

Let $\mu$ be a non-negative Radon measure which satisfies (2.2) and $\textbf{H}_\mu$ be the operator generated by sesquilinear form (2.1). Assume that the negative part of the spectrum of $\textbf{H}_\mu$ is discrete. Denote by $(\lambda _n)_{n \in \mathbb {N}}$ the non-decreasing sequence of negative eigenvalues (counting multiplicities) with the convention that $\lambda _{m+1} = \lambda _{m+2} = \dots = \Lambda =\inf \sigma _{ess}(\textbf{H}_\mu )$ if there are only *m* eigenvalues below the lowest point $\Lambda$ of the essential spectrum. Then, for each $n \in \mathbb {N}$,

$$\begin{aligned}&\sup \left\{ - \lambda< 0: ~\mathcal {L}(\{ x: ~q_2^*(x) \ge \lambda /4\}) \le \frac{n-1}{\sqrt{\Vert q_2^*\Vert _\infty }}\right\} \\&\quad \le \lambda _n \\&\quad \le \inf \left\{ -\lambda < 0: ~\mathcal {L}(\{ x: ~q_\alpha ^*(x) \ge 4\lambda \}) \ge \frac{n}{\sqrt{\lambda }}\right\} , \end{aligned}$$

where $$\alpha = \left( \pi ^2 + 3/4\right) ^{-1}$$.

#### Proof

Suppose that $\lambda$ satisfies $\mathcal {L}(\{ x: ~q_2^*(x) \ge \lambda /4\}) \le \frac{n-1}{\sqrt{\Vert q_2^*\Vert _\infty }}$. Then, by Theorem 4.8,

$$\begin{aligned} N(-\lambda , \textbf{H}_\mu ) \le n-1, \end{aligned}$$

so that $\lambda _n \ge -\lambda$. On the other hand, if $-\lambda$ satisfies $\mathcal {L}(\{ x: ~q_\alpha ^*(x) \ge 4\lambda \}) \ge \frac{n}{\sqrt{\lambda }}$, then Theorem 4.8 yields $N(-\lambda , \textbf{H}_\mu ) \ge n$, hence $\lambda _n \le -\lambda$. $\square$

As corollary, we obtain a *lower* estimate for the number $N_-(\textbf{H}_{\mu })$ of negative eigenvalues of $\textbf{H}_{\mu }$.

#### Corollary 4.11

Under the above general conditions for the measure $\mu$,

4.11 $$\begin{aligned} N_-(\textbf{H}_{\mu }) \ge \sup _{\varepsilon > 0} \frac{\varepsilon ^{3/2}}{2} \int _{\{ q_\alpha ^*\ge \varepsilon \}} \frac{1}{q_\alpha ^*} dx, \end{aligned}$$

where $$\alpha = \left( \pi ^2 + 3/4\right) ^{-1}$$.

#### Proof

We use the conventions from Theorem 4.10. Let $N(0, \textbf{H}_\mu ) = n$. Then, $\lambda _{n+1} = 0$. Hence, for every $\lambda > 0$,

4.12 $$\begin{aligned} \frac{n+1}{\sqrt{\lambda }} > \mathcal {L}(\{ x: ~q_\alpha ^*(x) \ge 4\lambda \})\ge 4\lambda \int _{\{ q_\alpha ^*\ge 4\lambda \}} \frac{1}{q_\alpha ^*(x)}~\textrm{d}x. \end{aligned}$$

This yields for every $\varepsilon > 0$:

$$\begin{aligned} n+1 > \frac{\varepsilon ^{3/2}}{2} \int _{\{ q_\alpha ^*\ge \varepsilon \}} \frac{1}{q_\alpha ^*(x)} ~\textrm{d}x, \end{aligned}$$

just what we need. $\square$

In particular, from (4.11), one can immediately obtain a *necessary* condition for the finiteness of the negative spectrum.

#### Remark 4.12

In the results above, we essentially made use of the lower bounds obtained in Theorem 4.8. Since $q_\alpha (x)$ decays at infinity not faster than $|x|^{-2}$, the upper bound in this theorem tends to infinity as $\lambda \rightarrow 0$; therefore, it is useless for estimating the number of all negative eigenvalues from above.

We conclude this section with an estimate for the lowest point of the spectrum.

#### Corollary 4.13

Let $\mu$ be a non-negative Radon measure, as usual, and $\textbf{H}_\mu$ be the operator generated by sesquilinear form (2.1). Let $\lambda _1 = \inf \sigma (\textbf{H}_\mu )$. Then, the following estimates hold true:

$$\begin{aligned} -4\Vert q_2^*\Vert _{\infty } \le \lambda _1 \le -\frac{1}{4} \Vert q_{\alpha }^*\Vert _{\infty }, \end{aligned}$$

where $$\alpha = \left( \pi ^2 + 3/4\right) ^{-1}$$.

#### Proof

Recall that $\lambda _1 = \sup \{ \lambda \in \mathbb {R}: ~N(\lambda , H_{\mu }) = 0\}$. Assume that $-\lambda < -4\Vert q_2^*\Vert _\infty$, i.e., $\lambda /4 > \Vert q_2^*\Vert _\infty$. Then, by Theorem 4.8,

$$\begin{aligned} N(-\lambda , \textbf{H}_\mu ) \le \sqrt{\Vert q_2^*\Vert _\infty } \mathcal {L}( \{ x: ~q_2^*(x) \ge \lambda /4\}). \end{aligned}$$

By our assumption on $\lambda$, $\{ x: ~q_2^*(x) \ge \lambda /4\} = \emptyset$, and therefore $N(-\lambda , \textbf{H}_\mu ) \le 0$, as required.

On the other hand, if $-\lambda > -\Vert q_{\alpha }^*\Vert _\infty /4$, that is, $4\lambda < \Vert q_{\alpha }^*\Vert _\infty$, then

$$\begin{aligned} N(-\lambda , \textbf{H}_{\mu }) \ge \sqrt{\lambda } \mathcal {L}(\{ x: ~q_{\alpha }^*(x) \ge 4\lambda \}). \end{aligned}$$

Since $4\lambda < \Vert q_{\alpha }^*\Vert _\infty$, the set $\{ x: ~q_{\alpha }^*(x) \ge 4\lambda \}$ is non-empty. Since $q_{\alpha }^*$ is continuous, the set needs to have a positive Lebesgue measure. Hence, $N(-\lambda , \textbf{H}_{\mu })>0$ and, being integer it is not less than 1. This means that $\lambda _1 \le -\lambda$. $\square$

Note that in Corollary 4.13, it is not supposed that the negative spectrum of $\textbf{H}_\mu$ is discrete or that the lowest point of the spectrum is an eigenvalue. An estimate for the lowest point of the essential spectrum can be obtained as well.

#### Corollary 4.14

Under the above conditions, let $\Lambda = \inf \sigma _{ess}(\textbf{H}_\mu )$. We set

$$\begin{aligned} Q_1 = \limsup _{x \rightarrow \pm \infty } q_2^*(x), \qquad Q_2 = \liminf _{x \rightarrow \pm \infty } q_\alpha ^*(x), \end{aligned}$$

where $$\alpha = (\pi ^2 + 3/4)^{-1}$$. Then,

$$\begin{aligned} -4Q_1 \le \Lambda \le -\frac{1}{4} Q_2. \end{aligned}$$

#### Proof

Recall that $\Lambda = \sup \{ \lambda \in \mathbb {R}: ~N(\lambda , \textbf{H}_{\mu }) < \infty \}$. Assume that $-\lambda < -4Q_1$, or equivalently $\lambda /4 > Q_1$. Then, by Theorem 4.8, we have

$$\begin{aligned} N(-\lambda , \textbf{H}_{\mu }) \le \sqrt{\Vert q_2^*\Vert _\infty } \mathcal {L}(\{ x: ~q_2^*(x) \ge \lambda /4\}). \end{aligned}$$

By the assumption on $\lambda$, the Lebesgue measure above is finite. Therefore, we have $N(-\lambda , \textbf{H}_{\mu }) < \infty$, i.e., $\Lambda \ge -\lambda$.

If $-\lambda > -Q_2/4$, then $4\lambda < Q_2$. Thus,

$$\begin{aligned} N(-\lambda , \textbf{H}_{\mu }) \ge \sqrt{\lambda } \mathcal {L}(\{ x: ~q_\alpha ^*(x) \ge 4\lambda \}) = \infty , \end{aligned}$$

since the set on the right-hand side contains at least one infinite interval $(-\infty , a)$ or $(b, \infty )$. Thus, $\Lambda \le -\lambda$. $\square$

### Lieb–Thirring Type Estimates

Now we are ready to establish the **LT** estimates for any $\gamma > 0$ for an arbitrary Radon measure $\mu$. Moreover, we prove lower estimates for $\textbf{LT}_{\gamma }$ for all $\gamma > 0$; in the special case $\gamma = \frac{1}{2}$, the lower bound can be expressed explicitly in terms of $\mu$ itself. For other values of $\gamma$, our estimates are expressed via the Otelbaev’s function. Throughout this section, we assume that the negative part of the spectrum of $\textbf{H}_{\mu }$ is discrete, that is, $q_1^*(x)\rightarrow 0 \quad$ as $x\rightarrow \pm \infty$ according to Lemma 3.9 and Remark 2.4.

We recall that for $\gamma >0$,

4.13 $$\begin{aligned} \textrm{LT}_{\gamma }(\textbf{H}_{\mu })\equiv \sum _{\nu =1}^\infty |\lambda _{\nu }|^\gamma = \gamma \int _{-\infty }^{0} |\lambda |^{\gamma - 1} N(\lambda ,\textbf{H}_\mu ) ~d\lambda , \end{aligned}$$

as soon as one of the quantities in (4.13) is finite.

We now establish a reverse **LT** estimate for $\gamma = 1/2$. For completeness, the following theorem presents our result (a lower bound) alongside a known result (an upper bound) from [17].

#### Theorem 4.15

Let $\mu$ be a non-negative Radon measure satisfying the Brinck condition (2.2). Let $\textbf{H}_\mu$ be the operator associated with the sesquilinear form (2.1). Assume that the negative part of the spectrum of $\textbf{H}_\mu$ is discrete. Let $\{\lambda _\nu \}$ be the negative eigenvalues of $\textbf{H}_\mu$, then

4.14 $$\begin{aligned} \frac{\alpha }{32} \mu (\mathbb {R})\le \sum _{\nu =1}^\infty |\lambda _\nu |^{\frac{1}{2}} \le \frac{1}{2}\mu (\mathbb {R}), \end{aligned}$$

where $$\alpha = \left( \pi ^2 + 3/4\right) ^{-1}$$.

The theorem should be understood in the sense that all the occurring terms in (4.14) are either simultaneously finite or infinite.

#### Proof

The upper bound was proved in [17]. Let us prove the lower bound. By (4.5),

$$\begin{aligned} \sum _{\nu =1}^\infty |\lambda _\nu |^\gamma&\ge \gamma \int _{-\infty }^{0} |\lambda |^{\gamma - 1} \sqrt{|\lambda |} \mathcal {L} \left( \left\{ x\in \mathbb {R}: q^*_{\alpha }(x) \ge 4|\lambda |\right\} \right) ~d\lambda \\&\ge \gamma \int _{-\infty }^{0} |\lambda |^{\gamma - \frac{1}{2}} \int _{\mathbb {R}} \chi _{\left\{ x\in \mathbb {R}: q^*_{\alpha }(x) \ge 4|\lambda |\right\} }(y)~\textrm{d}y~d\lambda , \end{aligned}$$

where $\chi _A$ is the indicator function of the set *A*. Using Fubini’s theorem, we obtain

4.15 $$\begin{aligned} \sum _{\nu =1}^\infty |\lambda _\nu |^\gamma \ge \gamma \int _{\mathbb {R}}\int _{-\frac{1}{4}q_\alpha ^*(x)}^0 |\lambda |^{\gamma - \frac{1}{2}}~d\lambda ~\textrm{d}x = \frac{\gamma }{\gamma + \frac{1}{2}} \left( \frac{1}{4}\right) ^{\gamma +\frac{1}{2}} \int _{\mathbb {R}} \left( q_\alpha ^*(x)\right) ^{\gamma +\frac{1}{2}} ~\textrm{d}x. \end{aligned}$$

We now set $\gamma = 1/2$. Let $I_k$ again be the intervals from Sect. 4.1 and $x_k$ be their middle points. Then

$$\begin{aligned} \sum _{\nu =1}^\infty |\lambda _\nu |^{\frac{1}{2}} \ge \frac{1}{8} \sum _{k \in \mathbb {K}} \int _{I_k} q_\alpha ^*(x)~\textrm{d}x \ge \frac{1}{8}\sum _{k \in \mathbb {K}: ~\mu _k(I_k) > 0} \int _{I_k} q_\alpha ^*(x)~\textrm{d}x. \end{aligned}$$

Lemma 3.6 implies now

$$\begin{aligned} \sum _{\nu =1}^\infty |\lambda _\nu |^{\frac{1}{2}} \ge \frac{1}{32} \sum _{k \in \mathbb {K}: ~\mu _k(I_k) > 0} |I_k| q_\alpha ^*(x_k). \end{aligned}$$

Using $q_\alpha ^*(x_k) = \frac{1}{d_\alpha (x_k)^2}$, Lemma 4.3 yields:

$$\begin{aligned} \sum _{\nu =1}^\infty |\lambda _\nu |^{\frac{1}{2}} \ge \frac{1}{32} \sum _{k \in \mathbb {K}: ~\mu _k(I_k)> 0} |I_k| \frac{1}{|I_k|^2} \ge \frac{\alpha }{32} \sum _{k \in \mathbb {K}: ~\mu _k(I_k) > 0} \mu _k(I_k) = \frac{\alpha }{32} \mu (\mathbb {R}). \end{aligned}$$

This finishes the proof. $\square$

For a general $\gamma >0$, the classical Lieb–Thirring estimate, where the potential is raised to the power of $\frac{1}{2} + \gamma$, seems not to have a reasonable analogy for a measure-potential, unlike $\gamma =\frac{1}{2}$. Here, we establish a Lieb–Thirring type estimate expressed in terms of the function $q_\alpha ^*$. We want to emphasize that the following result covers the subcritical case $0< \gamma < \frac{1}{2}$ as well.

#### Theorem 4.16

Let $\mu$ be a non-negative Radon measure satisfying (2.2) and $\textbf{H}_\mu$ be the operator generated by quadratic form (2.1). Suppose that the negative part of the spectrum of $\textbf{H}_\mu$ is discrete. Let $\gamma >0$ and let $\{\lambda _\nu \}$ be the negative eigenvalues of $\textbf{H}_\mu$, then

$$\begin{aligned} \frac{\gamma }{\gamma + \frac{1}{2}} \left( \frac{1}{4}\right) ^{\gamma +\frac{1}{2}} \int _{\mathbb {R}} |q_{\alpha }^*(x)|^{\frac{1}{2} + \gamma } dx \le \sum _{\nu =1}^\infty |\lambda _\nu |^\gamma \le 4^{\gamma +1} \int _{\mathbb {R}} \left| q_2^*(x)\right| ^{ \frac{1}{2} + \gamma }dx \end{aligned}$$

if the integrals converge (they do this simultaneously). Otherwise, $\sum _{\nu =1}^\infty |\lambda _\nu |^\gamma = \infty$. Here, $\alpha = \left( \pi ^2 + 3/4\right) ^{-1}$.

#### Proof

The lower bound follows from (4.15). We now derive the upper bound. By using (4.13) and the upper bound in Theorem 4.8, one sees that

$$\begin{aligned} \sum _{\nu =1}^\infty |\lambda _\nu |^\gamma \le 4\gamma \int _{-\infty }^{0} |\lambda |^{\gamma - 1} \int _{\left\{ x\in \mathbb {R}: q_2^*(x)\ge \frac{|\lambda |}{4}\right\} } \sqrt{q_2^*(y)}~\textrm{d}y ~d\lambda . \end{aligned}$$

Using Fubini’s theorem, we derive

$$\begin{aligned} \sum _{\nu =1}^\infty |\lambda _ \nu |^\gamma \le 4\gamma \int _{\mathbb {R}} \sqrt{q_2^*(x)} \int _{-4q_2^*(x)}^0 |\lambda |^{\gamma - 1}d\lambda dx = 4^{\gamma +1} \int _{\mathbb {R}} \left( q_2^*(x)\right) ^{\gamma + \frac{1}{2}}dx. \end{aligned}$$

This yields the required estimate. $\square$

This theorem and Lemma 3.10 show:

#### Theorem 4.17

Let $\mu$ be a positive Radon measure satisfying (2.3). Then, for every $\gamma > 0$, the following holds true:

$$\begin{aligned} C_1 \int _{\mathbb {R}} \left| q_{1}^*(x)\right| ^{ \frac{1}{2} + \gamma }~\textrm{d}x \le \sum _{\nu =1}^\infty |\lambda _\nu |^\gamma \le C_2 \int _{\mathbb {R}} \left| q_{1}^*(x)\right| ^{ \frac{1}{2} + \gamma }~\textrm{d}x, \end{aligned}$$

where

$$\begin{aligned} C_1 = \frac{\gamma }{\gamma + \frac{1}{2}} \left( \frac{1}{2\pi ^2 + \frac{3}{2}} \right) ^{1 + 2\gamma }, \qquad C_2 = 2^{4\gamma + 3}. \end{aligned}$$

Next, we estimate $\textbf{LT}_\gamma (\textbf{H}_\mu )$ in terms of the intervals $I_k$ from Sect. 4.1 with respect to the constant $\alpha = 2$

#### Theorem 4.18

Let $\mu$ be a non-negative Radon measure satisfying (2.3). Let $\gamma >0$ and $\{\lambda _\nu \}$ be the negative eigenvalues of $\textbf{H}_\mu$. Then, the following holds true:

$$\begin{aligned} \sum _{\nu =1}^\infty |\lambda _\nu |^\gamma \le 2^{2\gamma } \sum _{k\in \mathbb {K}} \mu _k(I_k)^{2\gamma }. \end{aligned}$$

#### Proof

In notations of Lemma 4.7, we have

$$\begin{aligned} \sum _{\nu =1}^\infty |\lambda _\nu |^\gamma \le \gamma \int _{-\infty }^{0} |\lambda |^{\gamma - 1} \left( \sum _{k \in \mathbb {K}:~-\lambda \ge -\frac{1}{|I_k|^2}} 1\right) ~d\lambda . \end{aligned}$$

We will use use the following notation for the shifted Heaviside function:

$$\begin{aligned} \mathbb {H}_{-\frac{1}{|I_k|^2}}(-\lambda ) := {\left\{ \begin{array}{ll} 1 &  \text {if } -\lambda \ge -\frac{1}{|I_k|^2},\\ 0 &  \text {otherwise.} \end{array}\right. } \end{aligned}$$

Then, the last estimate can be rewritten as follows:

$$\begin{aligned} \sum _{\nu =1}^\infty |\lambda _\nu |^\gamma&\le \gamma \int _{-\infty }^{0} |\lambda |^{\gamma - 1} \sum _{k\in \mathbb {K}} \mathbb {H}_{-\frac{1}{|I_k|^2}}(-\lambda ) ~d\lambda \le \sum _{k\in \mathbb {K}} \gamma \int _{-\infty }^{0} |\lambda |^{\gamma - 1} \mathbb {H}_{-\frac{1}{|I_k|^2}}(-\lambda ) ~d\lambda \\&= \sum _{k\in \mathbb {K}} \gamma \int _{-\frac{1}{|I_k|^2}}^{0} |\lambda |^{\gamma - 1} d\lambda = \sum _{k\in \mathbb {K}} \frac{1}{|I_k|^{2\gamma }}. \end{aligned}$$

Therefore, due to Lemma 4.3 with $\alpha =2$, we have proven the result. $\square$

## Data Availability

The authors declare that the data supporting the findings of this study are available within the paper.

## Appendix A. Comparison with Preceding Results

Here, we recall some known results and compare them with Theorem 4.16. Throughout this section, we assume that $q \in L_{\text {loc}}^1(\mathbb {R})$ is a non-negative function satisfying

A. 1 $$\begin{aligned} \int _{x}^{x+a} q(x)~\textrm{d}x \rightarrow 0 \quad \text {as} \quad |x|\rightarrow \infty \end{aligned}$$

for any fixed $a > 0$. Let $\mu$ be the measure defined by

A. 2 $$\begin{aligned} \mu (A) = \int _{A}q(x)dx \end{aligned}$$

for every Borel set $A\subset \mathbb {R}$. Due to (A. 1), the negative part of the spectrum of $\textbf{H}_\mu$ consists of negative eigenvalues $\{\lambda _\nu (q)\}$.

### A.1. Comparison with the Lieb–Thirring Estimate

We assume that $\gamma > 1/2$ and use notation $p=1/2 + \gamma$. Since the measure associated with *q* is continuous (no point masses), the following result holds:

#### Proposition A.1

Let $\alpha >0$ and $q \in L_{\text {loc}}^1(\mathbb {R})$ be a non-negative function. Then,

$$\begin{aligned} q_\alpha ^*(x) = \frac{\alpha }{d_\alpha (x)} \int _{x - d_\alpha (x)/2}^{x + d_\alpha (x)/2} q(y)dy. \end{aligned}$$

#### Proof

For a fixed $x\in \mathbb {R}$, consider two monotonous functions of the variable $d\in (0,\infty )$:

$$\begin{aligned} f_x(d): = \int _{x-d/2}^{x+d/2} q(y)dy, \qquad h_x(d): = \frac{1}{\alpha d}. \end{aligned}$$

These functions are continuous in *d*; moreover, the second one is strictly decaying. For *d* small enough, $f_x(d) < h_x(d)$, while for *d* large enough, $f_x(d) > h_x(d)$. Thus, there exists a unique solution $d_x$ to the equation $f_x(d_x) = h_x(d_x)$. By the definition of $q_\alpha ^*$, we have $q_\alpha ^*(x) = 1/d_x^2$, that is $d_\alpha (x) = d_x$. Then,

$$\begin{aligned} q_\alpha ^*(x) = \frac{1}{d_\alpha (x)^2} = \frac{\alpha }{d_\alpha (x)} \int _{x - d_\alpha (x)/2}^{x + d_\alpha (x)/2} q(y)~\textrm{d}y. \end{aligned}$$

This completes the proof. $\square$

The following result immediately follows from the classical Lieb–Thirring estimate, Lemma 3.6 and the lower bound in Theorem 4.16. However, we would like to give an alternative proof.

#### Theorem A.2

Let $\alpha >0$, $1<p<\infty$, then there exists $C_{p,\alpha }>0$ depending only on *p* and $\alpha$ such that for any non-negative function $q\in L^{p}(\mathbb {R})$, the estimate holds

$$\begin{aligned} \Vert q_{\alpha ,\mu }^*\Vert _{L^{p}(\mathbb {R})} \le C_{p,\alpha } \Vert q\Vert _{L^{p}(\mathbb {R})}. \end{aligned}$$

#### Proof

We recall the definition of the Hardy–Littlewood maximal function:

$$\begin{aligned} Mf(x) = \sup _{r>0} \frac{1}{|B(x,r)|} \int _{B(x,r)} |f(x)| dx. \end{aligned}$$

Using Proposition A.1, we obtain the estimate $q_\alpha ^*(x) \le \alpha Mq(x)$. Therefore, using the Hardy–Littlewood maximal inequality, we derive

$$\begin{aligned} \Vert q_{\alpha ,\mu }^*\Vert _{L^{p}(\mathbb {R})} \le \alpha \Vert Mq\Vert _{L^p(\mathbb {R})} \le \alpha C_{p} \Vert q\Vert _{L^{p}(\mathbb {R})}. \end{aligned}$$

This completes the proof. $\square$

The converse estimate does not hold. Consider the following example. Let $p>1$ and $x_k$ be the center point of $\mathcal {F}_{k}^+ = [2^{k-1}, 2^k]$, for $k\in \mathbb {N}$. We define

$$\begin{aligned} q(x) = {\left\{ \begin{array}{ll} 2^{\frac{k}{p}}, \quad & x \in \left( x_k - \frac{1}{2^{k + 1}}, x_k + \frac{1}{2^{k + 1}} \right) \text { and } k \text { is even},\\ 0, \quad &  \text {otherwise}. \end{array}\right. } \end{aligned}$$

#### Proposition A.3

Let $\alpha >0$ and $1<p<\infty$. Then, for $q\in L_{loc}^1(\mathbb {R})$, just defined,

$$\begin{aligned} \Vert q\Vert _{L^p(\mathbb {R})} = \infty \quad \text {and} \quad \Vert q_{\alpha ,\mu }^*\Vert _{L^p(\mathbb {R})} < \infty , \end{aligned}$$

where $\mu$ is the measure given by (A. 2).

#### Proof

Due to Lemma 3.6, it is enough to prove this for $\alpha =1$. For our function *q*, we clearly have $\Vert q\Vert _{L^p(\mathcal {F}_k^+)} = 1$ for an even *k*. Hence, we obtain the first statement.

To show the second statement, we write

A. 3 $$\begin{aligned} \Vert q_{1,\mu }^*\Vert _{L^p(\mathbb {R})}^p = \Vert q_{1,\mu }^*\Vert _{L^p((-\infty ,1))}^p + \sum _{k=2l-1; \; l\in \mathbb {N}} \Vert q_{1,\mu }^*\Vert _{L^p(\mathcal {F}_k^+)}^p + \sum _{k=2l; \; l\in \mathbb {N}} \Vert q_{1,\mu }^*\Vert _{L^p(\mathcal {F}_k^+)}^p. \end{aligned}$$

Since

A. 4 $$\begin{aligned} q_{1,\mu }^*(x) \le \frac{1}{\textrm{dist}(x, \textrm{supp}(q))^2}, \end{aligned}$$

we have

$$\begin{aligned} q_{1,\mu }^*(x) \le \frac{1}{(2-x)^{2}} \quad \text {for} \quad x\in (-\infty ,1), \end{aligned}$$

and hence, $$ \Vert q_{1,\mu }^*\Vert _{L^p((-\infty ,1))}<\infty $$.

Next, we assume that *k* is odd. Then $\textrm{dist}(\mathcal {F}_{k}^+, \textrm{supp}(q)) \asymp 2^k.$ Therefore, by (A. 4),

$$ \Vert q_{1,\mu }^*\Vert _{L^p(\mathcal {F}_k^+)}^p \asymp \frac{1}{2^{k(2p-1)}},$$

and since $p>1$, the second term on the right-hand side of (A. 3) is finite.

It remains to show that the third term on the right-hand side of (A. 3) is finite; thus, *k* is even. We will estimate $q_{1,\mu }^*$ on the following two subsets of $\mathcal {F}_k^+$:

A. 5 $$\begin{aligned} I_k = \left( 2^{k-1}, x_k - \frac{d_{1,\mu }(x_k)}{2} + \frac{1}{2^{k+1}} \right) , \qquad J_k = \left( x_k - \frac{d_{1,\mu }(x_k)}{2} + \frac{1}{2^{k+1}}, x_k \right) . \end{aligned}$$

This gives the estimate of $L^p$-norm of $q_{1,\mu }^*$ over $[2^{k-1},x_k]\subset \mathcal {F}_k^+$. The remaining part of $\mathcal {F}_k^+$, the set $[x_k, 2^k]$, can be treated similarly. We first integrate $(q_{1,\mu }^*)^p$ over $J_k$. Since $p>1$, we know that $2^{k(1 - 1/p) - 1} > 1/2^{k+1}$ for sufficiently large *k*. Therefore,

$$\begin{aligned} \mu \left( \Delta _{x_k} \left( 2^{k \left( 1- \frac{1}{p}\right) } \right) \right) = \int _{x_k- 2^{k(1 - 1/p) - 1}}^{x_k + 2^{k(1 - 1/p) - 1}} q(y) ~\textrm{d}y = \frac{1}{2^{k \left( 1- \frac{1}{p}\right) }}. \end{aligned}$$

We conclude that $d_{1,\mu }(x_k) = 2^{k \left( 1- \frac{1}{p}\right) }$, see Remark 3.2. Let $x\in J_k$. Then,

$$\begin{aligned} x - \frac{d_{1,\mu }(x_k) }{2}< x_k - \frac{1}{2^{k+1}}, \qquad x_k + \frac{1}{2^{k+1}} < x +\frac{d_{1,\mu }(x_k) }{2}, \end{aligned}$$

for sufficiently large *k*. Therefore,

$$\begin{aligned} \mu \left( \Delta _{x} \left( d_{1,\mu }(x_k) \right) \right) = \int _{x - \frac{d_{1,\mu }(x_k) }{2} }^{x + \frac{d_{1,\mu }(x_k) }{2} } q(y) ~\textrm{d}y = \int _{x_k- \frac{1}{2^{k+1}}}^{x_k + \frac{1}{2^{k+1}}} 2^{\frac{k}{p}} ~\textrm{d}y = \frac{1}{d_{1,\mu }(x_k)}. \end{aligned}$$

By Remark 3.2, $d_{1,\mu }\arrowvert _{J_k} = 2^{k(1 - 1/p)}$ and

A. 6 $$\begin{aligned} \Vert q_{1,\mu }^*\Vert _{L^p(J_k)}^p = \frac{1}{2^{k\left( 2p - 3 +\frac{1}{p}\right) }}. \end{aligned}$$

Next, we evaluate $q_{1,\mu }^*$ for $x\in I_k$: since

$$\begin{aligned} \textrm{dist}(x,\textrm{supp}(q)) = \textrm{dist}\left( x, \left[ x^k -\frac{1}{2^{k+1}},x^k +\frac{1}{2^{k+1}}\right] \right) . \end{aligned}$$

(A. 4) implies:

$$\begin{aligned} q_{1,\mu }^*(x) \le \frac{1}{\left( x_k - \frac{1}{2^{k+1}} - x\right) ^2}, \qquad \text {for } x\in I_k, \end{aligned}$$

and hence,

$$\begin{aligned} \Vert q_{1,\mu }^*\Vert _{L^p(I_k)}^p \lesssim \frac{1}{2^{k\left( 2p - 3 +\frac{1}{p}\right) }}. \end{aligned}$$

Combining this with (A. 6), we obtain the above estimate for the interval $[2^{k-1},x_k]$. By the similar arguments, we study $q_{1,\mu }^*$ on $[x_k,2^k]$ and obtain

$$\begin{aligned} \Vert q_{1,\mu }^*\Vert _{L^p(\mathcal {F}_k^+)}^p \lesssim \frac{1}{2^{k\left( 2p - 3 +\frac{1}{p}\right) }}. \end{aligned}$$

Since $p>1$, $2p - 3 +\frac{1}{p} > 0$, therefore, the third term on the right-hand side of (A. 3) is finite, and hence, $\Vert q_{1,\mu }^*\Vert _{L^p(\mathbb {R})}$ is finite. $\square$

#### Remark A.4

The above results show that our version of the Lieb–Thirring estimate yields finite bounds for absolutely continuous potentials as long as the classical Lieb–Thirring estimate does. But for the potential *q* in the above example, our bound gives a finite bound, while the classical Lieb–Thirring estimate yields no information.

### A.2. Comparison with the Subcritical Netrusov–Weidl Estimate

We now consider the subcritical case, that is, $0<\gamma <1/2$. For $q\in L_{\text {loc}}^1(\mathbb {R})$, we define

$$\begin{aligned} A_\gamma (q) = \sum _{k=0}^{\infty } \left( \int _{\mathcal {F}_k} (1+|x|)^\sigma q(x)dx\right) ^{\frac{1}{2}+\gamma } + \left( \sum _{k=0}^{\infty } \left( \int _{\mathcal {F}_k} (1+|x|)^\sigma q(x) dx\right) ^{\frac{1}{2}+\gamma }\right) ^{\frac{2\gamma }{\frac{1}{2} + \gamma }}, \end{aligned}$$

$$\begin{aligned} B_\gamma (q)= \sum _{k=0}^{\infty } \left( \int _{k}^{k+1} q(x)dx\right) ^{2\gamma } + \sum _{k=0}^\infty \left( \int _{k}^{k+1} q(x)dx\right) ^{\frac{1}{2}+\gamma }, \end{aligned}$$

where $\sigma = \frac{1/2 - \gamma }{1/2 + \gamma }$. We recall the following essential result; see [23]:

#### Theorem A.5

(Netrusov, Weidl). Let $0<\gamma <1/2$ and $\sigma = \frac{1/2 - \gamma }{1/2 + \gamma }$. Define the sets $\mathcal {F}_0 = [-1,1]$ and $\mathcal {F}_k = [-2^k,-2^{k-1}]\cup [2^{k-1},2^k]$, for $k\in \mathbb {N}.$ Then, there exists $C>0$ depending only on $\gamma$ such that

$$\begin{aligned} \sum _{\nu \in \mathbb {N}} |\lambda _\nu (q)|^\gamma \le C \min \left\{ A_\gamma (q), B_\gamma (q)\right\} , \quad \text {for any } q\in L_{\text {loc}}^1(\mathbb {R}). \end{aligned}$$

#### Remark A.6

Here, we stated special versions of Theorems 3 and 4 from [23] for the one-dimensional, non-fractional case.

Next, we note that the combination of Theorem A.5, the lower bound in Theorem 4.16, and Lemma 3.10 gives the following estimate:

#### Corollary A.7

Let $\alpha >0$, and $\gamma$, $\sigma$, $\mathcal {F}_k$, $A_\gamma (q)$, $B_\gamma (q)$ be as in Theorem A.5. Then, there exists $C>0$ depending only on $\alpha$ and $\gamma$ such that

$$\begin{aligned} \int _{\mathbb {R}} |q_{\alpha ,\mu }^*(x)|^{\frac{1}{2} + \gamma } dx \le C \min \left\{ A_\gamma (q), B_\gamma (q)\right\} , \end{aligned}$$

for any $q\in L_{\text {loc}}^1(\mathbb {R})$ satisfying (A. 1), where $\mu$ is the measure given by (A. 2).

The converse relation does not hold; see the construction below.

#### Example A.8

Let $$0<\gamma <1/2$$, $$\sigma = \frac{1/2 - \gamma }{1/2 + \gamma }$$, and $$x_k$$ be the center point of $$ \mathcal {F}_{k}^+ = [2^{k-1}, 2^k]$$. Define a function $$ q = q_1 + q_2$$, where

$$\begin{aligned} q_1(x)&= {\left\{ \begin{array}{ll} 1, \quad & x \in \left( x_k - \frac{1}{2^{k\sigma + 1}}, x_k + \frac{1}{2^{k\sigma + 1}} \right) \text { and } k \text { is even},\\ 0 &  \text {otherwise}, \end{array}\right. }\\ q_2(x)&= {\left\{ \begin{array}{ll} \frac{2^3}{2^{\frac{k}{2\gamma }}} &  x \in \left( x_k - 2^{k-3}, x_k + 2^{k-3} \right) \text { and } k \text { is even},\\ 0 &  \text {otherwise}. \end{array}\right. } \end{aligned}$$

We show that for the example above, the upper bounds in Theorem A.5 are infinite, while the upper bound in Theorem 4.16 is finite.

#### Proposition A.9

Let $\alpha >0$, then there exists $q\in L_{loc}^1(\mathbb {R})$ such that

$$\begin{aligned} A_\gamma (q) = \infty , \qquad B_\gamma (q) = \infty , \qquad \Vert q_{1,\mu }^*\Vert _{L^{\frac{1}{2}+\gamma }(\mathbb {R})}<\infty , \end{aligned}$$

where $\mu$ is the measure given by (A. 2).

#### Proof

Due to Lemma 3.6, it suffices to prove the statement for $\alpha =1$ only. Let *q* be the function defined in Example A.8. For an even *k*, it is clear that

$$\begin{aligned} \int _{2^{k-1}}^{2^k} (1 + x)^\sigma q_1(x) ~\textrm{d}x \gtrsim 1, \qquad \sum _{m=2^{k-1}}^{2^{k}-1} \left( \int _m^{m+1} q_2(x) ~\textrm{d}x\right) ^{2\gamma } \gtrsim 1. \end{aligned}$$

Therefore, the first two statements are true.

Let $\mu _1$ and $\mu _2$ be the measures defined by (A. 2) with *q* replaced by $q_1$ and $q_2$, respectively. Then, by Lemma 3.11, we know that

$$\begin{aligned} \Vert q_{1,\mu }^*\Vert _{L^{\frac{1}{2}+\gamma }(\mathbb {R})} \lesssim \Vert q_{1,\mu _1}^*\Vert _{L^{\frac{1}{2}+\gamma }(\mathbb {R})} + \Vert q_{1,\mu _2}^*\Vert _{L^{\frac{1}{2}+\gamma }(\mathbb {R})}. \end{aligned}$$

We write, for $m=1,2$,

A. 7 $$\begin{aligned}  &   \Vert q_{1,\mu _m}^*\Vert _{L^{\frac{1}{2}+\gamma }(\mathbb {R})}^{\frac{1}{2}+\gamma }= \Vert q_{1,\mu _m}^*\Vert _{L^{\frac{1}{2}+\gamma }((-\infty ,1))}^{\frac{1}{2}+\gamma }+ \sum _{k=2l-1; \; l\in \mathbb {N}} \Vert q_{1,\mu _m}^*\Vert _{L^{\frac{1}{2}+\gamma }(\mathcal {F}_k^+)}^{\frac{1}{2}+\gamma }\nonumber \\  &   \quad + \sum _{k=2l; \; l\in \mathbb {N}} \Vert q_{1,\mu _m}^*\Vert _{L^{\frac{1}{2}+\gamma }(\mathcal {F}_k^+)}^{\frac{1}{2}+\gamma }. \end{aligned}$$

By the same arguments we do in the proof of Proposition A.3, the first term on the right-hand side is finite for $m=1,2.$

To investigate the second term, we consider odd *k* first. For $m=1,2$, we estimate

$$\begin{aligned} \textrm{dist}(\mathcal {F}_k^+, \textrm{supp}(q_m)) \le \textrm{dist}(\mathcal {F}_k^+, \textrm{supp}(q)) \asymp 2^k. \end{aligned}$$

Hence, (A. 4) implies

$$\begin{aligned} \Vert q_{1,\mu _m}^*\Vert _{L^{\frac{1}{2}+\gamma }(\mathcal {F}_k^+)}^{\frac{1}{2}+\gamma } \asymp 2^{k-1} \frac{1}{(2^{2k})^{\frac{1}{2} + \gamma }} \asymp \frac{1}{2^{2k\gamma }}. \end{aligned}$$

Therefore, the second term in (A. 7) on the right-hand side of (A. 7)is finite.

It remains to show that the third term is finite for $m=1,2$, this means, for an even *k*. We consider the potential $q_{1,\mu _1}$ on the following subsets in $\mathcal {F}_k^+$:

$$\begin{aligned} I_k^1 = \left( 2^{k-1}, x_k - \frac{d_{1,\mu _1}(x_k)}{2} + \frac{1}{2^{k\sigma +1}}\right) , \qquad J_k^1 = \left( x_k - \frac{d_{1,\mu _1}(x_k)}{2} + \frac{1}{2^{k\sigma +1}}, x_k \right) . \end{aligned}$$

We study $q_{1,\mu }^*$ in $J_k^1$ first. Since $0<\sigma <1$, for sufficiently large *k*,

$$\begin{aligned} \left( x_k - \frac{1}{2^{k\sigma + 1}}, x_k + \frac{1}{2^{k\sigma + 1}} \right) \subset \left( x_k - 2^{k\sigma - 1}, x_k + 2^{k\sigma - 1}\right) \subset \mathcal {F}_k^+. \end{aligned}$$

Thus, $\mu _1(\Delta _{x_k}(2^{k\sigma })) = {2^{-k\sigma }},$ and hence, $d_{1,\mu _1}(x_k) = 2^{k\sigma }$, see Remark 3.2. For $x\in J_k^1$,

$$\begin{aligned} x - 2^{k\sigma - 1}< x_k -\frac{1}{2^{k\sigma +1}}, \qquad x_k +\frac{1}{2^{k\sigma +1}} < x + \frac{d_{1,\mu _1}(x_k)}{2} = x + 2^{k\sigma - 1} \end{aligned}$$

and hence, $\mu _1(\Delta _{x} (2^{k\sigma })) =2^{-k\sigma }$. Therefore, as in Remark 3.2, $d_{1,\mu _1}\arrowvert _{J_k^1} = 2^{k\sigma }$, so that

$$\begin{aligned} \Vert q_{1,\mu _1}^*\Vert _{L^{\frac{1}{2}+\gamma }(J_k^1)}^{{\frac{1}{2}+\gamma }} = \left( \frac{d_{1,\mu _1}(x_k)}{2} - \frac{1}{2^{k\sigma +1}}\right) \frac{1}{2^{2k\sigma \left( \frac{1}{2} + \gamma \right) }} \asymp \frac{1}{2^{2k\sigma \gamma }}. \end{aligned}$$

Let $$x\in I_k^1$$, then

A. 8 $$\begin{aligned} \textrm{dist}(x,\textrm{supp}(q_1)) = x_k - \frac{1}{2^{k\sigma + 1}} - x. \end{aligned}$$

Now, (A. 4) implies

$$\begin{aligned} \Vert q_{1,\mu _1}^*\Vert _{L^{\frac{1}{2}+\gamma }(I_k^1)}^{\frac{1}{2}+\gamma } \le \int _{I_k^1} \frac{1}{\left( x_k - \frac{1}{2^{k\sigma + 1}} - x\right) ^{1+2\gamma }}~\textrm{d}x \le \frac{1}{\left( \frac{d_{1,\mu _1}(x_k)}{2} - \frac{1}{2^{k\sigma }} \right) ^{2\gamma }} \lesssim \frac{1}{2^{2k\sigma \gamma }}. \end{aligned}$$

Combining this with (A. 8), we obtain

$$\begin{aligned} \Vert q_{1,\mu _1}^*\Vert _{L^{\frac{1}{2}+\gamma }(2^{k-1},x_k)}^{\frac{1}{2}+\gamma } \lesssim \frac{1}{2^{2k\sigma \gamma }}. \end{aligned}$$

In the same way, we investigate $q_{1,\mu }^*$ on the interval $[x_k,2^k]$ and derive

$$\begin{aligned} \Vert q_{1,\mu _1}^*\Vert _{L^{\frac{1}{2}+\gamma }(\mathcal {F}_k^+)}^{\frac{1}{2}+\gamma } \lesssim \frac{1}{2^{2k\sigma \gamma }}. \end{aligned}$$

Since $\gamma <1/2$, the latter estimates show that the third term on the right-hand side in (A. 7) is finite for $m=1$. We will show that this is also true for $m=2$. To study $q_{1,\mu _2}^*$ on $\mathcal {F}_k^+$ with an even *k*, we will consider two cases: $\gamma \le 1/4$ and $\gamma >1/4$.

Let $$\gamma \le 1/4$$. For $$x\in \mathcal {F}_k^+$$,

$$\begin{aligned} (x - 2^{k-2}, x + 2^{k-2}) \subset \mathcal {F}_{k-1}^+ \cup \mathcal {F}_{k}^+\cup \mathcal {F}_{k+1}^+ \end{aligned}$$

and $q_{1,\mu _2}^*\arrowvert _{\mathcal {F}_{k-1}^+\cup \mathcal {F}_{k+1}^+} =0$. Therefore, using $\gamma \le 1/4$, we estimate

$$\begin{aligned} \int _{x - 2^{k-2}}^{x+2^{k-2}} q_2(y)~\textrm{d}y \le 2^{k-2} \frac{2^3}{2^{\frac{k}{2\gamma }}} \le \frac{1}{2^{k-1}} \end{aligned}$$

for sufficiently large *k*. Then, $d_{1,\mu _2}(x)>2^{k-1}$ for $x\in \mathcal {F}_k^+$ and therefore:

A. 9 $$\begin{aligned} \Vert q_{1,\mu _2}^*\Vert _{L^{\frac{1}{2} + \gamma }(\mathcal {F}_k^+)}^{ \frac{1}{2} + \gamma }\lesssim \frac{1}{2^{2k\gamma }}. \end{aligned}$$

Hence, the third term on the right-hand side of (A. 7) is finite for $m=2$.

Finally, for $$\gamma >1/4$$,

$$\begin{aligned} \int _{x_k - 2^{k-2}}^{x_k+2^{k-2}} q_2(x)\textrm{d}x = 2^{k-2} \frac{2^3}{2^{\frac{k}{2\gamma }}} \ge \frac{1}{2^{k-1}} \end{aligned}$$

for sufficiently large *k*, and hence, $d_{1,\mu _2}(x_k)< 2^{k-1}$. This means that for $x\in \mathcal {F}_k^+$,

$$\begin{aligned} \left( x - \frac{d_{1,\mu _2}(x_k)}{2}, x + \frac{d_{1,\mu _2}(x_k)}{2}\right) \subset \mathcal {F}_{k-1}^+ \cup \mathcal {F}_{k}^+\cup \mathcal {F}_{k+1}^+, \end{aligned}$$

for sufficiently large *k*. Therefore, since $q_{1,\mu _2}^*\arrowvert _{\mathcal {F}_{k-1}^+\cup \mathcal {F}_{k+1}^+} =0$, we estimate

$$\begin{aligned} \frac{1}{d_{1,\mu _2}(x)} = \int _{x-\frac{d_{1,\mu _2}(x)}{2}}^{x+\frac{d_{1,\mu _2}(x)}{2}} q_2(y)\textrm{d}y \lesssim d_{1,\mu _2}(x) \frac{1}{2^{\frac{k}{2\gamma }}}, \quad \text {for } x\in \mathcal {F}_k^+, \end{aligned}$$

so that $q_{1,\mu _2}^*(x) = 1/d_{1,\mu _2}^2(x)\lesssim 1/2^{\frac{k}{2\gamma }}$. Therefore,

$$\begin{aligned} \Vert q_{1,\mu _2}^*\Vert _{L^{\frac{1}{2} + \gamma }(\mathcal {F}_k^+)}^{ \frac{1}{2} + \gamma } \lesssim 2^k \frac{1}{2^{\frac{k}{2\gamma }(\frac{1}{2}+\gamma )}}. \end{aligned}$$

Since $\frac{1}{2\gamma } \left( \frac{1}{2} + \gamma \right) \ge 1$ for $\gamma <1/2$, we conclude from this and (A. 9) that for both $\gamma \le 1/4$ and $\gamma >1/4$, the third term on the right-hand side in (A. 7) is finite for $m=2$. This completes the proof. $\square$

#### Remark A.10

For *q* as in Example A.8, Theorem A.5 gives no information due to Proposition A.9. However, from Theorem 4.16 and Proposition A.9, we conclude that $\textrm{LT}_{\gamma }(\textbf{H}_\mu )$ is finite, which shows that our estimates are sharper.

## Appendix B. Examples

In this section, we obtain some corollaries and give some examples.

Let $(x_k)_{k \in \mathbb {Z}}$ be a sequence tending to $\pm \infty$ as *k* tends to $\pm \infty$. Further, let $(a_k)_{k \in \mathbb {Z}}$ be a sequence of positive numbers. Consider the measure $\mu =\sum _{k\in \mathbb {Z}} a_k\delta _{ x_k}$. Of course, the quadratic form $\textbf{a}_\mu$ is not lower-bounded when the sequence $(a_k)_{k \in \mathbb {Z}}$ is unbounded. Hence, we consider examples with case $a_k \rightarrow 0$ in the following.

### Corollary B.1

Let $\mu =\sum _{k=1}^\infty a_k\delta _{x_k}$. Assume that there exists $\alpha >0$ such that

$$\begin{aligned} x_{k+1} - x_k>\max \left\{ (\alpha a_k)^{-1}, (\alpha a_{k+1})^{-1} \right\} \end{aligned}$$

for all $k\in \mathbb {N}$. Then,

B.1 $$\begin{aligned} C_1 \alpha ^{2\gamma }\sum _{k=1}^\infty a_k^{2\gamma }\le \sum _{k=1}^\infty \lambda _k^\gamma \le C_2 \alpha ^{2\gamma } \sum _{k=1}^\infty a_k^{2\gamma } \end{aligned}$$

for some $C_1$, $C_2>0$ independent of $\{x_k\}$ and $\{a_k\}$.

### Proof

We note that

$$\begin{aligned} q_\alpha ^*(x)= \left\{ \begin{array}{lll} (a_k\alpha )^2,&  \text {if}&  x\in (x_k-(2a_k\alpha )^{-1},x_k+(2a_k\alpha )^{-1})\\ \left( \min \left\{ \frac{1}{x-x_k} ,\frac{1}{x_{k+1}-x} \right\} \right) ^2,&  \text {if}&  x\in (x_k+(2a_k\alpha )^{-1},x_{k+1}-(2a_{k+1}\alpha )^{-1}). \end{array} \right. \end{aligned}$$

Therefore, for $$k\in \mathbb {N}$$,

$$\begin{aligned} \int _{x_k-(2a_k\alpha )^{-1}}^{x_k+(2a_k\alpha )^{-1}}(q_\alpha ^*(x))^{\frac{1}{2}+\gamma }~\textrm{d}x = (a_k\alpha )^{2\gamma } \end{aligned}$$

and

$$\begin{aligned}  &   \int _{x_k+(2a_k\alpha )^{-1}}^{x_{k+1}-(2a_{k+1}\alpha )^{-1}}(q_\alpha ^*(x))^{\frac{1}{2}+\gamma }\textrm{d}x \le \int _{-\infty }^{x_{k+1}-(2a_{k+1}\alpha )^{-1}}\left( \frac{1}{x_{k+1}-x} \right) ^{1+2\gamma }\textrm{d}x\\  &   \quad + \int _{x_k+(2a_k\alpha )^{-1}}^\infty \left( \frac{1}{x-x_{k}} \right) ^{1+2\gamma }\textrm{d}x \asymp (a_{k+1}\alpha )^{2\gamma }+(a_k\alpha )^{2\gamma }. \end{aligned}$$

Consequently, we obtain (B.1). $\square$

We note again that for the classical Lieb–Thirring estimate (1.2), when the potential can be written as $V = V_1 + V_2$, with disjoint supports, the inequality does not reflect how far these two supports are apart from each other. This behavior is fundamentally different from our Lieb–Thirring type estimates. We demonstrate this with the following explicit example.

### Example B.2

Let $\mu = \delta _0 + \delta _y$, where $y > 0$. The Otelbaev function has different forms, depending on $y < \frac{1}{4}$, $\frac{1}{4} \le y < \frac{1}{2}$ or $y \ge \frac{1}{2}$. Explicit computations show the following: for $y \ge \frac{1}{2}$,

$$\begin{aligned} q_2^*(x) = {\left\{ \begin{array}{ll} \frac{1}{4x^2}, \quad & x \le -\frac{1}{4},\\ 4, \quad & - \frac{1}{4} \le x \le \frac{1}{4},\\ \frac{1}{4x^2}, \quad & \frac{1}{4} \le x \le \frac{y}{2},\\ \frac{1}{4(y-x)^2}, \quad & \frac{y}{2} \le x \le y -\frac{1}{4},\\ 4, \quad &  y-\frac{1}{4} \le x \le y + \frac{1}{4},\\ \frac{1}{4(y-x)^2}, \quad & x \ge y + \frac{1}{4}. \end{array}\right. } \end{aligned}$$

Next, for $\frac{1}{4} \le y < \frac{1}{2}$, Otelbaev’s function equals

$$\begin{aligned} q_2^*(x) = {\left\{ \begin{array}{ll} \frac{1}{4x^2}, \quad & x \le -\frac{1}{4},\\ 4, \quad &  -\frac{1}{4} \le x \le y - \frac{1}{4},\\ \frac{1}{4(y-x)^2}, \quad &  y - \frac{1}{4} \le x \le \frac{y}{2},\\ \frac{1}{4x^2}, \quad & \frac{y}{2} \le x \le \frac{1}{4},\\ 4, \quad &  \frac{1}{4}\le x \le y + \frac{1}{4},\\ \frac{1}{4(y-x)^2}, \quad & x \ge y + \frac{1}{4}. \end{array}\right. } \end{aligned}$$

Finally, for $$0< y < \frac{1}{4}$$,

$$\begin{aligned} q_2^*(x) = {\left\{ \begin{array}{ll} \frac{1}{4x^2}, \quad & x \le -\frac{1}{4},\\ 4, \quad & -\frac{1}{4} \le x \le y - \frac{1}{4}\\ \frac{1}{4(y-x)^2}, \quad &  y-\frac{1}{4} \le x \le y - \frac{1}{8}\\ 16, \quad &  y - \frac{1}{8} \le x \le \frac{1}{8}, \\ \frac{1}{4x^2}, \quad &  \frac{1}{8} \le x \le \frac{1}{4}, \\ 4, \quad &  \frac{1}{4} \le x \le y + \frac{1}{4},\\ \frac{1}{4(y-x)^2}, \quad & x \ge y + \frac{1}{4}. \end{array}\right. } \end{aligned}$$

Now, for $\gamma > 0$ we have the following expressions for the Lieb–Thirring estimates from Theorem 4.16: For $y \ge \frac{1}{2}$, it is

$$\begin{aligned} 4^{\gamma +1}\int _\mathbb {R} |q_2^*(x)|^{\gamma + \frac{1}{2}}~\textrm{d}x&= \frac{4\cdot 16^\gamma }{\gamma } + 8\cdot 16^\gamma - \frac{2\cdot 4^\gamma \cdot y^{-2\gamma }}{\gamma }. \end{aligned}$$

For $\frac{1}{4} \le y < \frac{1}{2}$, we have

$$\begin{aligned} 4^{\gamma +1}\int _\mathbb {R} |q_2^*(x)|^{\gamma + \frac{1}{2}}~\textrm{d}x&= \frac{\gamma 4^{2\gamma + 2}y+y^{-2\gamma }4^{\gamma + \frac{1}{2}}}{\gamma }; \end{aligned}$$

and for $0 < y \le \frac{1}{4}$, one sees that

$$\begin{aligned} 4^{\gamma + 1}\int _{\mathbb {R}} |q_2^*(x)|^{\gamma + \frac{1}{2}}~\textrm{d}x&= \frac{4^{3\gamma + \frac{1}{2}} + y\gamma 4^{2\gamma + 2} + \gamma 4^{3\gamma + \frac{5}{2}}(\frac{1}{8} - \frac{y}{2})}{\gamma }. \end{aligned}$$

This calculation shows the following interesting limit behavior for the upper Lieb–Thirring estimate, which is not captured by the classical Lieb–Thirring estimates: When the two components of $\mu$ move away from each other, we obtain the behavior:

$$\begin{aligned} 4^{\gamma + 1}\int _{\mathbb {R}} |q_2^*(x)|^{\gamma + \frac{1}{2}}~\textrm{d}x \overset{y \rightarrow \infty }{\longrightarrow }\frac{4\cdot 16^\gamma }{\gamma } + 8\cdot 16^\gamma . \end{aligned}$$

On the other hand, when the components become close to each other, we see that:

$$\begin{aligned} 4^{\gamma + 1}\int _{\mathbb {R}} |q_2^*(x)|^{\gamma + \frac{1}{2}}~\textrm{d}x \overset{y \rightarrow 0}{\longrightarrow }\ \frac{4^{3\gamma + \frac{1}{2}} + \gamma 4^{3\gamma + 1}}{\gamma }. \end{aligned}$$

Note that the limit $y \rightarrow 0$ agrees with the Lieb–Thirring bound obtained for the potential $\mu = 2\delta _0$: Indeed, for this potential,

$$\begin{aligned} q_2^*(x) = {\left\{ \begin{array}{ll} 16, \quad & |x|\le \frac{1}{8},\\ \frac{1}{4|x|^2}, \quad & |x| > \frac{1}{8}. \end{array}\right. } \end{aligned}$$

It is not hard to verify that, for this function, the integral $4^{\gamma + 1}\int _{\mathbb {R}}|q_2^*(x)|^{\gamma + \frac{1}{2}}~\textrm{d}x$ agrees with the above value. Of course, this is no coincidence, as it can easily be verified by the continuity result [16, Prop. III.5] and the dominated convergence theorem.

On the other hand, for the limit $y \rightarrow \infty$, one might expect to recover the estimates for the potential $\delta _0$. But this is indeed not the case. Note that Otelbaev’s function for the potential $\delta _0$ is given by:

$$\begin{aligned} q_2^*(x) = {\left\{ \begin{array}{ll} 4, \quad & |x| \le \frac{1}{4},\\ \frac{1}{4|x|^2}, \quad & |x| > \frac{1}{4}. \end{array}\right. } \end{aligned}$$

With this explicit form, it is simple to verify that

$$\begin{aligned} 4^{\gamma + 1} \int _{\mathbb {R}}|q_2^*(x)|^{\gamma + \frac{1}{2}}~\textrm{d}x&= \frac{4\gamma +2}{\gamma }4^{2\gamma }. \end{aligned}$$

This is clearly not the limit that we obtained for $y \rightarrow \infty$ above.

### Example B.3

Assume that the measure $\mu$ is compactly supported, say, $[-R,R] = \operatorname {conv}(\operatorname {supp}(\mu ))$, the convex hull of the support of $\mu$. Then, for any $|x| \ge R + \frac{1}{2\alpha \mu (R)}$,

$$\begin{aligned} q_\alpha ^*(x) \le \frac{1}{\operatorname {dist}^2(x, \operatorname {supp}(\mu ))} \le \frac{1}{(x - R)^2}. \end{aligned}$$

Also, for any $x\in \mathbb {R}$, by using Lemma 3.3, we estimate

$$\begin{aligned} \mu (\mathbb {R}) \ge \mu (\Delta _x(d_\alpha (x))) \ge \frac{1}{\alpha d_\alpha (x)} = \frac{1}{\alpha } \sqrt{q_\alpha ^*(x)}. \end{aligned}$$

Therefore,

$$\begin{aligned} \int _{\mathbb {R}}|q_\alpha ^*(x)|^{\frac{1}{2}+\gamma }\textrm{d}x&\le 2\int _0^{R + \frac{1}{2\alpha \mu (R)}} (\alpha \mu (\mathbb {R}))^{1+2\gamma }\textrm{d}x + 2\int _{R + \frac{1}{2\alpha \mu (R)}}^\infty \frac{1}{(x - R)^{1+2\gamma }}\textrm{d}x\\&\le R\alpha ^{1+2\gamma } \mu (\mathbb {R})^{1+2\gamma } + \alpha ^{2\gamma } \left( \frac{1}{2} + \frac{2^{2\gamma - 1}}{\gamma }\right) \mu (\mathbb {R})^{2\gamma }. \end{aligned}$$

Hence, due to Theorem 4.16,

$$\begin{aligned} \sum _{k=1}^\infty |\lambda _k|^\gamma \le 2^{3+4\gamma } R \mu (\mathbb {R})^{1+2\gamma } + 2^{4\gamma + 1} \left( 1 + \frac{2^{2\gamma }}{\gamma }\right) \mu (\mathbb {R})^{2\gamma }. \end{aligned}$$
